# MTC-TCN-Attn: A TCN-Attention-Based Multi-Task Collaborative Prediction Method for Wheat Soil Moisture

**DOI:** 10.3390/s26206471

**Published:** 2026-10-13

**Authors:** Weiguang Ning, Jiwei Gao, Taoyang Han, Xingrong Cong, Caixia Song

**Affiliations:** 1 Qingdao Smart Rural Development Service Center, Qingdao 266109, China; 2 College of Science and Information, Qingdao Agricultural University, Qingdao 266109, China; 3 College of Mechanical and Electrical Engineering, Qingdao Agricultural University, Qingdao 266109, China

**Keywords:** soil moisture prediction, Temporal Convolutional Network, self-attention mechanism, multi-task learning

## Abstract

Wheat is one of the most important staple crops worldwide, and soil moisture plays a critical role in regulating crop growth, irrigation management, and agricultural water-use efficiency. Accurate soil moisture prediction is therefore essential for precision agriculture and intelligent irrigation scheduling. However, soil moisture variations are driven by complex interactions among meteorological conditions, soil layers, and temporal dynamics, resulting in strong nonlinear characteristics and long-term dependencies that remain challenging for conventional prediction approaches. To address these challenges, this study proposes a Multi-Task Collaborative Temporal Convolutional Network with Attention (MTC-TCN-Attn) model for multi-variable wheat soil moisture prediction. The proposed framework integrates a Temporal Convolutional Network (TCN) with a multi-head self-attention mechanism, where causal and dilated convolutions are employed to capture multi-scale temporal dependencies, while the attention module adaptively identifies informative time steps and enhances global temporal feature representation. Furthermore, a multi-task collaborative learning strategy based on shared feature representations is developed to exploit the intrinsic correlations among multiple soil-moisture-related variables and improve model generalization. Experiments conducted on wheat field data demonstrate that the proposed MTC-TCN-Attn model outperforms representative benchmark models, including SVR, LSTM, Transformer, and Multi-Task Long Short-Term Memory (MT-LSTM), across all five prediction targets in terms of $R^{2}$, RMSE, and MAE. For the three principal soil-moisture-state variables, the proposed model achieved $R^{2}$ values of 0.9147, 0.9651, and 0.9622 for surface soil moisture (SSM), subsurface soil moisture (SUSM), and soil moisture profile (SMP), respectively. Compared with single-task and multi-task TCN baselines, the proposed MTC-TCN-Attn model consistently reduces prediction errors and improves robustness by effectively integrating temporal dependency modeling and cross-task information sharing. The results indicate that the proposed framework provides an effective approach for multi-variable soil moisture forecasting and has promising potential for intelligent irrigation management and precision agricultural applications.

## 1. Introduction

Wheat is one of the most important cereal crops worldwide, and it plays an important role in ensuring global food security [1]. Soil moisture is a key environmental factor affecting wheat growth, development, and yield formation, and is directly related to root development, nutrient transport, photosynthesis, and grain formation [2,3,4,5,6]. With increasing pressure on agricultural water resources and the growing demand for precision irrigation, accurate monitoring and prediction of soil moisture dynamics are important for optimizing irrigation scheduling, improving water-use efficiency, and supporting precision agricultural management [7,8,9].

Soil moisture dynamics are jointly influenced by precipitation, temperature, evapotranspiration, antecedent moisture conditions, and moisture variations across different soil layers, resulting in pronounced nonlinear, temporal, and lagged characteristics [10,11,12]. Traditional physically based models can describe soil water transport processes from a mechanistic perspective, but they generally require substantial prior knowledge, numerous environmental parameters, and model calibration, which may limit their application under complex agricultural conditions [13,14]. With the development of data-driven approaches, machine learning and deep learning methods have increasingly been applied to soil moisture prediction and have demonstrated strong capabilities in modeling complex nonlinear relationships and temporal features [15,16,17].

Among existing deep learning approaches, recurrent neural networks (RNNs), long short-term memory (LSTM) networks, gated recurrent units (GRUs), and Transformer-based models have been widely applied to time-series prediction of soil moisture and related environmental variables [17]. However, these models still exhibit certain limitations in long-sequence modeling efficiency, the utilization of historical information, and information sharing among multiple related prediction targets. In particular, for multi-variate soil moisture prediction involving both cross-variable relationships and complex temporal dependencies, determining how to effectively exploit complementary information among different targets within a unified model while identifying the historical temporal features that are more informative for the current prediction remains an important issue to be addressed [18].

More specifically, three issues remain to be addressed in current soil moisture prediction studies. First, many existing methods formulate soil moisture forecasting as an independent single-target problem [19,20,21,22]. Such a strategy does not explicitly exploit the complementary information and statistical dependence among multiple soil moisture variables. In this study, these variables comprise SSM, SUSM, SMP, SSMA, and SUSMA, which characterize moisture conditions at the surface, subsurface/root-zone, and whole-profile levels, together with their corresponding anomaly indicators. Consequently, potentially useful cross-variable information is not fully utilized during representation learning.

Second, although multi-task learning provides a natural mechanism for sharing information among related prediction targets, simple shared representations do not necessarily benefit all tasks equally [23,24]. Different soil-moisture-related variables exhibit distinct statistical distributions and temporal dynamics. Direct parameter sharing may therefore introduce task interference when a common representation cannot adequately accommodate heterogeneous target characteristics [24,25]. An effective multi-task framework should not only exploit cross-variable correlations but also maintain sufficient temporal representation capability for different prediction targets.

Third, Temporal Convolutional Networks (TCNs) can efficiently capture long-range temporal dependencies through causal and dilated convolutions and enable parallel computation [26]. However, convolution-based temporal modeling primarily learns dependencies through fixed convolutional parameters and does not explicitly distinguish the varying importance of historical time steps [26,27]. This limitation is particularly relevant to soil moisture dynamics, because precipitation, evapotranspiration, and temperature variations may exert nonuniform and time-varying lagged effects on current soil water conditions. Therefore, effectively identifying informative historical observations while preserving multi-scale temporal dependencies remains an important problem [27].

Accordingly, the key problem addressed in this study is determining how to jointly exploit cross-variable dependencies and nonuniform temporal dependencies within a unified prediction framework for multiple heterogeneous soil-moisture-related targets. To address this problem, we propose a Multi-Task Collaborative Temporal Convolutional Network with Attention (MTC-TCN-Attn), which integrates TCN-based temporal modeling [26], multi-head self-attention [27,28], and multi-task learning [23] within a unified prediction framework. The proposed framework employs a shared TCN encoder to extract multi-scale temporal features from meteorological and soil-moisture sequences, while causal and dilated convolutions preserve temporal causality and enlarge the receptive field. A multi-head self-attention mechanism is subsequently introduced to adaptively evaluate the importance of different historical time steps and enhance global temporal feature representation. Meanwhile, the multi-task learning strategy enables related prediction targets to share temporal representations and complementary information within a unified model.

The main contributions of this study are summarized as follows:

1. A unified MTC-TCN-Attn framework is developed for the simultaneous prediction of five heterogeneous soil-moisture-related variables, including surface soil moisture (SSM), subsurface soil moisture (SUSM), soil moisture profile (SMP), surface soil moisture anomaly (SSMA), and subsurface soil moisture anomaly (SUSMA). Unlike conventional independent single-target prediction, the proposed framework combines shared multi-task representation learning with TCN-based multi-scale temporal feature extraction, allowing cross-variable dependencies to be exploited within a single prediction model.
2. A multi-head self-attention mechanism is incorporated after the shared TCN feature extractor to complement the fixed temporal weighting characteristics of convolutional modeling. The TCN is responsible for extracting local and multi-scale causal temporal patterns, whereas self-attention adaptively identifies informative historical time steps and models global temporal dependencies. To separately evaluate the effects of multi-task parameter sharing and temporal attention, a controlled comparison chain consisting of a Single-Task TCN, a Multi-Task TCN without attention, and the complete MTC-TCN-Attn model is established.
3. The proposed framework is quantitatively evaluated through internal model comparisons, representative machine learning and deep learning benchmarks, and temporal-robustness experiments. MTC-TCN-Attn achieves $R^{2}$ values of 0.9147, 0.9651, and 0.9622 for SSM, SUSM, and SMP, respectively. Compared with the Single-Task TCN, the RMSE values of these three principal soil-moisture variables are reduced by approximately 12.7%, 4.9%, and 4.9%, respectively, demonstrating the effectiveness of the proposed framework for joint prediction of multiple soil moisture variables.

## 2. Related Work

Soil moisture (SM) is an important variable for characterizing land-surface water conditions. Its dynamics are jointly affected by multiple factors, including precipitation, temperature, evapotranspiration, soil properties, vegetation cover, and antecedent moisture conditions, and therefore exhibit pronounced nonlinearity, temporal lag effects, and multi-scale temporal variability [10]. Research on soil moisture prediction has gradually progressed from traditional machine learning (ML) methods to deep temporal modeling, attention-based architectures, and multi-task learning (MTL) [17]. These approaches have improved nonlinear relationship modeling, long-term temporal dependency extraction, and information sharing among related prediction tasks, respectively. However, for multiple soil-moisture-related variables that are mutually correlated but exhibit different statistical characteristics, determining how to effectively exploit both their temporal dependencies and intervariable relationships remains an important research problem [18].

Traditional ML methods were among the earliest data-driven approaches applied to soil moisture estimation and prediction. Support vector machine (SVM), random forest (RF), gradient boosting methods, and ensemble learning have been widely investigated because they can establish nonlinear mappings between soil moisture and meteorological, soil, and remote-sensing variables, while remaining applicable to scenarios with relatively limited sample sizes. Acharya et al. [10] developed ML models using soil properties, crop information, and nearby weather-station data to predict field soil moisture in the Red River Valley, where the SVM model captured nonlinear relationships between soil moisture, cumulative precipitation, and potential evapotranspiration. Karthikeyan and Mishra [12] employed ML methods for multi-layer high-resolution soil moisture estimation across the United States and achieved an unbiased root mean square error (ubRMSE) below 0.04 m^3^/m^3^ at most validation sites. For vertical extrapolation of surface soil moisture to deeper layers under alpine freeze–thaw conditions, Li and Zhang [11] compared several prediction methods and showed that an SVM with a radial basis function (RBF) kernel outperformed linear approaches under complex freeze–thaw conditions. In addition, Yin et al. [29] and Huang et al. [30] optimized SVM parameters using the Arithmetic Optimization Algorithm (AOA) and Bald Eagle Search (BES) algorithm, respectively, to improve soil moisture prediction in tea plantations. These studies demonstrate that kernel-based methods and their optimized variants are effective in modeling complex nonlinear relationships between environmental variables and soil moisture.

In addition to SVM-based approaches, tree-based and ensemble learning methods have also been extensively applied to soil moisture prediction. By aggregating multiple decision trees, RF reduces the sensitivity of a single model to noise and sample perturbations, making it suitable for high-dimensional datasets containing meteorological, soil, and vegetation variables. Carranza et al. [31] incorporated multi-layer soil moisture observations and environmental variables into an RF framework for root-zone soil moisture (RZSM) estimation, demonstrating the capability of ensemble tree models to describe complex relationships among moisture conditions at different depths. Chen et al. [32] applied ML methods to soil moisture estimation during the winter wheat growing season and incorporated Synthetic Aperture Radar (SAR) observations to improve soil moisture retrieval. Gradient Boosting Machine (GBM), eXtreme Gradient Boosting (XGBoost), and stacking methods have further been used to improve model generalization by combining multiple learners. Lamichhane et al. [33] integrated Sentinel-1 SAR observations, Harmonized Landsat Sentinel (HLS) optical data, and texture features for soil moisture prediction, with the GBM model achieving favorable surface soil moisture estimation performance in a semi-arid agricultural region. Granata et al. [34] employed a stacked ML framework for multi-step-ahead soil moisture prediction, while Jamei et al. [35] investigated long-term multi-step forecasting of root-zone soil moisture under different climatic conditions using ensemble-based data-intelligent models. Although ensemble learning improves nonlinear fitting capability and robustness to noise, temporal information is generally represented through manually constructed features such as lagged variables or cumulative precipitation, limiting its ability to automatically learn representations from continuous historical sequences.

With the increasing availability of continuous meteorological observations, remote-sensing products, and ground-sensor measurements, deep learning models capable of directly processing sequential information have been increasingly adopted for soil moisture prediction. Long short-term memory (LSTM) networks regulate the retention and updating of historical information through gating mechanisms and have therefore been widely used for temporal soil moisture modeling. Abbes et al. [19] combined soil moisture active passive (SMAP) observations with an LSTM model for soil moisture estimation, demonstrating the ability of recurrent neural networks to learn dynamic patterns from continuous remote-sensing time series. Gao et al. [36] employed deep bidirectional LSTM networks to predict soil moisture and soil electrical conductivity in orchards. Zhao et al. [37] combined seasonal-trend decomposition using Loess (STL) with a Bidirectional Gated Recurrent Unit–Long Short-Term Memory (BiGRU-LSTM) model to characterize trend, periodic, and short-term variations in soil moisture over the Qinghai–Tibet Plateau. Beyond individual recurrent architectures, convolutional and recurrent networks have also been combined to jointly model local patterns and temporal dependencies. CNN–LSTM and related convolutional–recurrent architectures have therefore been explored for soil moisture prediction, with convolutional operations used for feature extraction and recurrent components used for sequential modeling [17,38].

As the prediction window increases, however, modeling long sequences solely through recurrent state propagation becomes more difficult. Consequently, convolution-based sequence modeling has also been introduced into environmental time-series prediction. The Temporal Convolutional Network (TCN) enlarges its temporal receptive field through causal and dilated convolutions and employs residual connections to improve the training stability of deep networks [26]. Unlike recurrent architectures that update hidden states sequentially across time steps, TCNs can extract temporal features in parallel while preserving temporal causality. Amamou et al. [39] combined a TCN with a gated recurrent unit (GRU) to construct an NRBO-TCN-GRU model for soil moisture forecasting at horizons of 1, 7, 15, and 30 days. In their framework, the TCN was used to extract temporal features at different scales, the GRU further modeled sequential dependencies, and the Newton–Raphson-Based Optimizer (NRBO) was employed for hyperparameter optimization. The results showed stable predictive performance across multiple forecasting horizons. These studies indicate that dilated convolution can effectively enlarge the historical receptive field of soil moisture prediction models [26]. Nevertheless, convolution-based sequence modeling mainly aggregates temporal information through learned local convolution kernels, and differences in the importance of individual historical time steps are generally represented only implicitly [27].

To further emphasize temporal positions or input variables that contribute more strongly to prediction, attention mechanisms have increasingly been incorporated into soil moisture prediction models. Li et al. [27] proposed an attention-aware LSTM model for soil moisture and soil temperature prediction, in which different predictors and temporal information were assigned adaptive weights to improve the identification of informative inputs. Kara et al. [40] combined a genetic algorithm (GA) with an attention-based deep learning model to optimize model architecture and parameters for soil moisture prediction. Han et al. [22] developed a Convolutional Attention Encoder–Decoder Long Short-Term Memory (CAEDLSTM) model that integrated convolutional feature extraction, an encoder–decoder architecture, and attention mechanisms to enhance soil moisture prediction under complex climatic conditions. Li et al. [41] further proposed the REDF-LSTM model by integrating residual encoder–decoder structures with feature attention, enabling adaptive selection of input variables according to their relevance to prediction. A comprehensive comparison of deep learning approaches also showed that different attention mechanisms do not provide uniform improvements in soil moisture prediction; the effectiveness of feature attention and temporal attention depends on the prediction task, input variables, and underlying model architecture [17]. Therefore, although attention mechanisms improve the model’s ability to identify informative features and time steps, their effectiveness remains dependent on the characteristics of the prediction target and the temporal representations produced by the underlying model.

Beyond improving feature representations for individual prediction tasks, recent studies have increasingly investigated how information can be shared among multiple related prediction targets. Soil moisture variables measured or represented at different temporal scales, soil layers, or moisture states are often correlated, whereas conventional single-task models optimize each target independently and cannot explicitly exploit potentially useful information shared among related targets. Multi-task learning (MTL) provides a mechanism for jointly optimizing multiple related tasks by sharing model parameters or feature representations [23]. In soil moisture forecasting, Baldi et al. [42] applied multi-task neural networks to vineyard Internet of Things (IoT) sensor data and formulated soil moisture values at different future forecasting horizons as related tasks. Their results showed that joint learning improved 24–48 h multi-step soil moisture forecasts compared with independent prediction, demonstrating the potential benefit of sharing information among different forecasting horizons.

Applications of MTL to soil moisture prediction have subsequently expanded from different forecasting horizons of the same variable to the joint modeling of different environmental variables and soil depths. Li et al. [18] proposed an Adaptive-Weight Long Short-Term Memory (AW-LSTM) model that jointly modeled soil moisture with soil temperature, skin temperature, surface latent heat flux, and surface sensible heat flux. The model employed dynamic loss weighting together with intertask correlation information to balance the optimization of different tasks. Their results showed that multi-task training could exploit information from related variables to improve soil moisture prediction, while differences in task relatedness and numerical scales affected the joint optimization process. Liu et al. [43] used leaf water content as an auxiliary task and jointly modeled soil moisture at depths of 5, 10, 20, and 40 cm. Their results indicated that multi-task learning mainly improved estimation at depths of 10–40 cm, whereas a comparable benefit was not observed for surface soil moisture at 5 cm. These findings further indicate that the relationships between auxiliary variables or different soil depths and the target variable are not uniform, and that the benefits of parameter sharing are strongly task dependent.

Existing multi-task soil moisture studies therefore highlight two important issues. On the one hand, knowledge sharing among related tasks can reduce the information isolation associated with independent modeling. In particular, when different forecasting horizons, soil depths, or related environmental variables exhibit strong dependencies, multi-task learning can exploit their complementary information. On the other hand, different tasks do not necessarily follow identical temporal dynamics. Soil moisture state variables, moisture conditions at different depths, and auxiliary environmental variables may differ in variation magnitude, response speed, and temporal dependency structure. When all tasks rely on the same shared representation, information that benefits one task may not be equally suitable for another, and differences in task relatedness or optimization directions may lead to negative transfer during shared learning [24,25]. Multi-task soil moisture prediction therefore requires consideration of both shared information and task heterogeneity, rather than simply increasing the number of jointly predicted targets.

Overall, existing studies have improved soil moisture prediction from several complementary perspectives. Traditional ML and ensemble learning methods can effectively establish nonlinear mappings between multi-source environmental variables and soil moisture, but their ability to automatically extract continuous temporal dependencies remains relatively limited [16]. Deep temporal models such as LSTM, GRU, and TCN improve historical dependency modeling; in particular, dilated causal convolutions in TCNs facilitate the extraction of temporal features at different scales, although the importance of individual historical positions is mainly encoded implicitly within convolutional representations [26]. Attention mechanisms provide an additional means of adaptively identifying informative variables and time steps, but their effectiveness varies with the prediction task and underlying temporal architecture [17]. MTL further enables information sharing among related prediction targets; however, existing soil moisture studies have primarily focused on different forecasting horizons of the same variable, different soil depths, or auxiliary environmental and physiological variables [43]. For prediction problems involving soil-moisture-related variables with heterogeneous statistical characteristics, including surface moisture, root-zone moisture, overall profile states, and their corresponding anomaly indicators, considering temporal dependencies alone or applying simple task sharing may be insufficient to fully characterize their dynamic relationships. Determining how to jointly account for multi-scale historical information, nonuniform contributions of different historical time steps, and shared relationships among multiple related prediction targets therefore remains an important problem in multi-variable soil moisture prediction.

## 3. Data and Methods

### 3.1. Study Area

**Figure 1:**
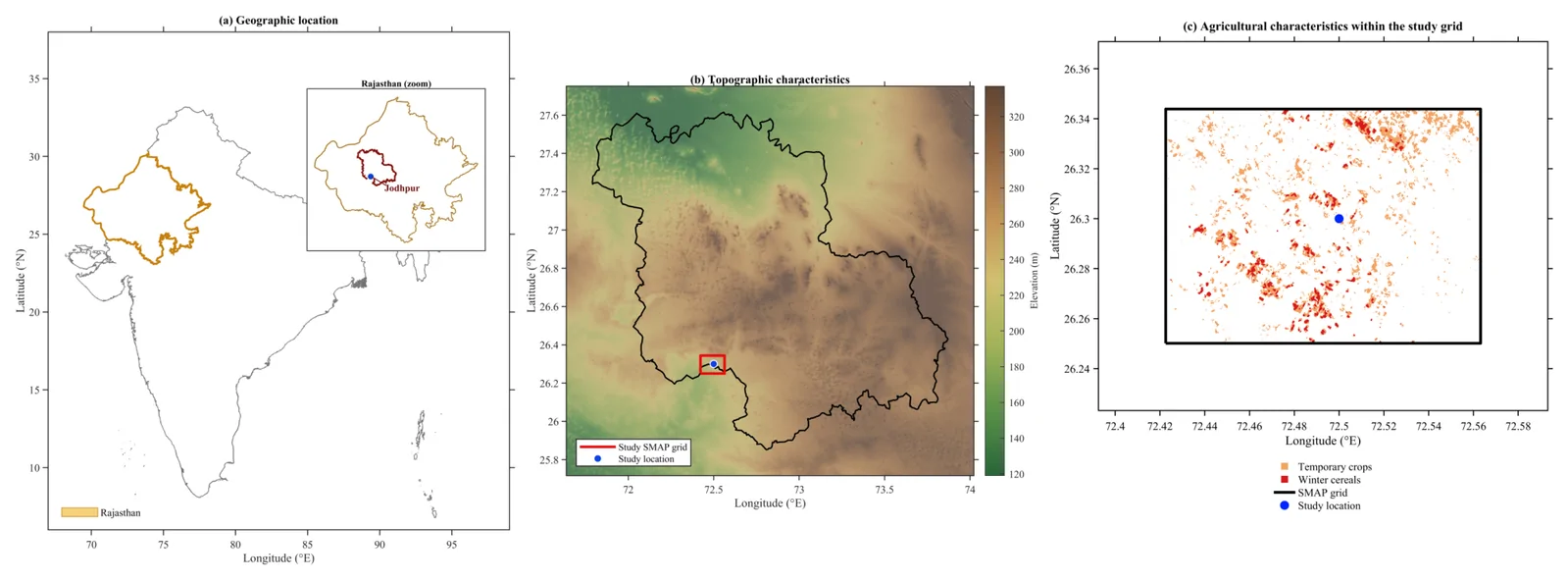
Study area and environmental characteristics. (**a**) Geographic location of the study area in Jodhpur, Rajasthan, India.The red outline indicates the boundary of Jodhpur, and the blue dot marks the study location. (**b**) Topographic characteristics of Jodhpur and the location of the study SMAP grid based on SRTM elevation data. (**c**) Agricultural characteristics within the study SMAP grid based on the ESA WorldCereal 2021 product. Temporary crops and winter cereals are shown together with the study grid and study location.

The study area is located in Jodhpur, Rajasthan, India, with the study location at $26.3^{\circ }$ N, $72.5^{\circ }$ E. According to the spatial grid of the NASA–USDA Enhanced SMAP product, the grid corresponding to the study location covers approximately $72.4226^{\circ }$–$72.5632^{\circ }$ E and $26.2501^{\circ }$–$26.3439^{\circ }$ N, with an area of approximately $146.14\;{\mathrm{km}}^{2}$. The geographical location, topographic characteristics, and agricultural land-use features of the study area are shown in Figure 1.

Jodhpur is located in the arid western part of Rajasthan and mainly falls within the Arid Western Plain (I-A) agro-climatic zone. The region is characterized by a dry climate, relatively low and highly variable annual precipitation, with annual rainfall ranging from approximately 216 to 496mm, as well as high summer temperatures and strong evaporation. The landscape is dominated by plains, sandy surfaces, and localized aeolian landforms, and the overall terrain is relatively flat. According to the SRTM digital elevation model, the elevation of Jodhpur and its surrounding areas shows noticeable spatial variation, while the study grid is located in a relatively low-lying area in southwestern Jodhpur, as shown in Figure 1b. The soils are mainly desert soils and sandy soils, which, together with the arid climatic conditions, form a typical soil-moisture environment of dryland regions.

Agriculture is an important land-use type in this region. The major Rabi-season crops in the agro-climatic zone containing Jodhpur include wheat, mustard, and cumin. To further characterize the agricultural distribution within the study grid, the ESA WorldCereal 2021 crop classification product was used for spatial analysis. The results show that approximately $13.61\;{\mathrm{km}}^{2}$ within the study grid was identified as temporary crops, accounting for approximately 9.31% of the total grid area. Of this area, approximately $2.64\;{\mathrm{km}}^{2}$ was identified as winter cereals, accounting for approximately 19.38% of the temporary-crop area, as shown in Figure 1c. Meanwhile, agricultural statistics from Rajasthan show that the wheat cultivation area in Jodhpur reached 77,939, 75,032, and 83,065ha in 2016–2017, 2019–2020, and 2020–2021, respectively. In addition, the SPAM2020 crop distribution dataset provides a nonzero wheat area for the grid containing the study location. These results indicate that the study area has a clear wheat-growing background, consistent with the agricultural soil-moisture prediction scenario considered in this study.

**Figure 2:**
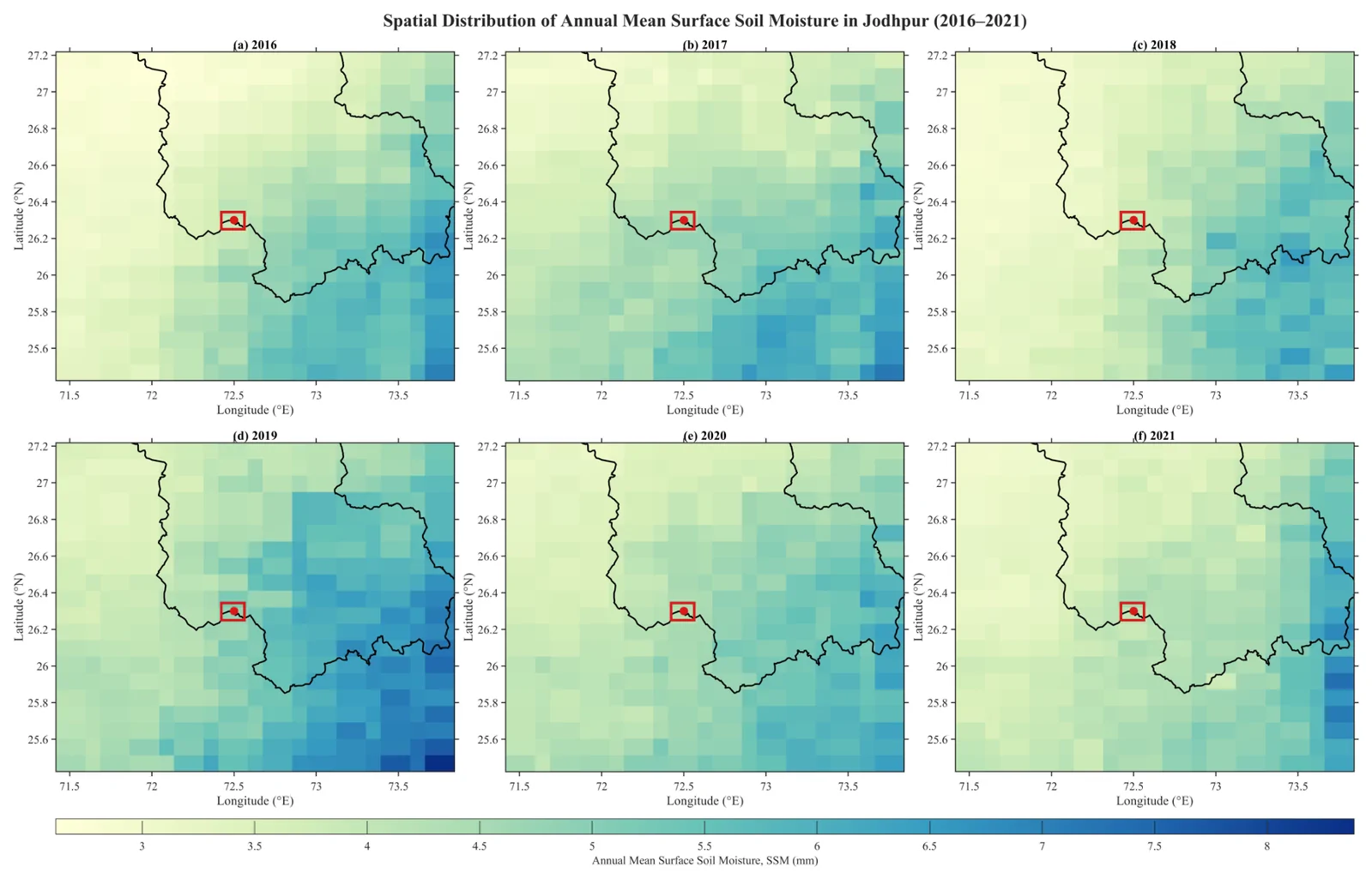
Spatial distributions of annual mean surface soil moisture (SSM) in the Jodhpur region from 2016 to 2021. The same spatial extent and color scale are used for all years. The red box and red point indicate the study SMAP grid and study location, respectively.

To further illustrate the spatial characteristics of soil moisture in the study area, the annual mean surface soil moisture (SSM) distributions from 2016 to 2021 were calculated using the NASA–USDA Enhanced SMAP data, as shown in Figure 2. A consistent spatial extent and color scale were used for all years to facilitate direct comparison. Overall, SSM shows clear spatial variability across the study area. The eastern and southeastern parts generally exhibit higher annual mean soil moisture, whereas relatively lower values are observed in the western and northwestern parts. The overall spatial pattern remains relatively consistent among different years, although the magnitude of soil moisture varies to some extent from year to year. The study grid is highlighted by the red box in each panel, providing a direct representation of its location within the regional spatial pattern of soil moisture.

### 3.2. Sample Extraction

#### 3.2.1. Data Sources and Variables

The dataset used in this study was constructed by integrating meteorological data from the NASA Prediction Of Worldwide Energy Resources (NASA POWER) project with soil-moisture data from the NASA–USDA Enhanced SMAP Global Soil Moisture Data product. Data were extracted for a grid location in the Jodhpur region of Rajasthan, India ($26.3^{\circ }$ N, $72.5^{\circ }$ E), covering the period from 1 January 2016 to 31 December 2021. After temporal harmonization and date alignment, the final dataset contained 2192 consecutive daily records.

The meteorological variables were obtained from the NASA POWER daily data service. The meteorological parameters provided by NASA POWER are primarily derived from the Modern-Era Retrospective Analysis for Research and Applications, Version 2 (MERRA-2), produced by the NASA Global Modeling and Assimilation Office (GMAO). The native spatial resolution of the MERRA-2 meteorological fields is $0.5^{\circ }\times 0.625^{\circ }$, and the values represent grid-cell-scale meteorological conditions rather than point-scale ground-station measurements. Five NASA POWER variables were used as meteorological inputs in this study: 2 m dew point temperature (T2MDEW), corrected precipitation (PRECTOTCORR), 10 m wind direction (WD10M), Earth skin temperature (TS), and 2 m air temperature (T2M).

The soil-moisture variables were obtained from the NASA–USDA Enhanced SMAP Global Soil Moisture Data product available in the Google Earth Engine (GEE) data catalog (dataset ID: NASA_USDA/HSL/SMAP10KM_soil_moisture). This product provides global soil-moisture information at a spatial resolution of 10 km and has a nominal temporal cadence of 3 days. It is generated by assimilating satellite-derived soil moisture active passive (SMAP) soil-moisture observations into a modified two-layer Palmer soil water balance model using a one-dimensional ensemble Kalman filter (EnKF). The Palmer model consists of a surface layer and a subsurface/root-zone layer. The surface layer has a maximum water-holding capacity of 25.4 mm, whereas the water-storage capacity of the subsurface/root-zone layer varies according to soil properties. Therefore, surface soil moisture (SSM) and subsurface soil moisture (SUSM) in this product represent modeled water storage in the two Palmer model layers rather than direct measurements at fixed geometric soil depths.

The definitions, data sources, roles, and units of the variables used in the prediction model are summarized in Table 1. SSM and SUSM were used as both input features and prediction targets, whereas soil moisture profile (SMP), surface soil moisture anomaly (SSMA), and subsurface soil moisture anomaly (SUSMA) were used only as prediction targets.

It should be emphasized that SMP is not equivalent to volumetric soil water content. Volumetric soil water content is conventionally defined as the ratio of the volume of water to the bulk volume of soil and is generally expressed in $m^{3}/m^{3}$. In contrast, SMP in the NASA–USDA Enhanced SMAP product is provided directly as the smp band and is expressed as a dimensionless fraction between approximately 0 and 1. It characterizes the relative moisture status of the entire modeled soil profile rather than the volumetric water content at a specific soil depth. Therefore, SMP was directly extracted from the NASA–USDA product in this study, and no conversion from SSM or SUSM to volumetric soil water content was performed.

SSMA and SUSMA are also not direct soil-moisture measurements at additional soil depths. Instead, they represent the standardized anomalies of SSM and SUSM, respectively. In the NASA–USDA Enhanced SMAP product, the anomaly is calculated relative to the climatological soil-moisture condition for the corresponding calendar period. The climatology is estimated using the available SMAP record with a 31-day centered moving window. A standardized soil-moisture anomaly can be generally expressed as

(1) $$A_{t}=\frac{X_{t}-\mu_{d}}{\sigma_{d}},$$

where $A_{t}$ denotes the standardized soil-moisture anomaly at time *t*, $X_{t}$ is the corresponding soil-moisture value, and $\mu_{d}$ and $\sigma_{d}$ denote the climatological mean and standard deviation for the corresponding calendar period, respectively. When $X_{t}$ represents SSM, $A_{t}$ corresponds to SSMA; when $X_{t}$ represents SUSM, $A_{t}$ corresponds to SUSMA. Positive anomaly values indicate wetter-than-climatological conditions, whereas negative values indicate drier-than-climatological conditions. In the present study, SSMA and SUSMA were directly obtained from the corresponding anomaly bands of the NASA–USDA Enhanced SMAP product rather than recalculated from SSM and SUSM. Equation (1) is provided to clarify the definition of the anomaly variables in the original data product.

Because the NASA POWER meteorological variables are available at a daily temporal resolution while the NASA–USDA Enhanced SMAP product has a nominal 3-day cadence, temporal harmonization was performed before merging the two data sources. Specifically, linear interpolation was applied between consecutive SMAP observations to convert SSM, SUSM, SMP, SSMA, and SUSMA to daily time series. For two consecutive original observations $x_{t}$ and $x_{t+3}$, the two intermediate daily values were calculated as

(2) $$x_{t+1}=x_{t}+\frac{1}{3}\left(x_{t+3}-x_{t}\right),$$

and

(3) $$x_{t+2}=x_{t}+\frac{2}{3}\left(x_{t+3}-x_{t}\right).$$

The resulting daily SMAP series was then aligned with the NASA POWER meteorological variables according to calendar date. This procedure produced a continuous daily dataset covering the complete study period. After temporal alignment, the merged dataset was checked for residual missing entries. In the model implementation, if any residual missing values were detected, they were replaced using the previous valid observation before constructing the prediction samples.

#### 3.2.2. Data Standardization

To eliminate the risk of data leakage, Z-score standardization is applied to both the input features and the output targets, with the corresponding means and standard deviations estimated exclusively from the training set.

For input features:

(4) $${\tilde{x}}_{t}^{\left(f\right)}=\frac{x_{t}^{\left(f\right)}-\mu_{x}^{\left(f\right)}}{\sigma_{x}^{\left(f\right)}}$$

where $\mu_{x}^{\left(f\right)}$ and $\sigma_{x}^{\left(f\right)}$ are calculated exclusively from the raw time steps covered by the training input windows. No observations from the test period are used to estimate these statistics.

For output targets:

(5) $${\tilde{y}}_{t}^{\left(k\right)}=\frac{y_{t}^{\left(k\right)}-\mu_{y}^{\left(k\right)}}{\sigma_{y}^{\left(k\right)}}$$

where $\mu_{y}^{\left(k\right)}$ and $\sigma_{y}^{\left(k\right)}$ are calculated exclusively from the target time steps corresponding to the training samples. No test targets are used in estimating these statistics. The model prediction output is the normalized value $\hat{\tilde{y}}$, which is ultimately back-normalized as

(6) $$\hat{y}=\hat{\tilde{y}}⊙\sigma_{y}+\mu_{y}$$

where ⊙ denotes element-wise multiplication.

### 3.3. Temporal Convolutional Network

The Temporal Convolutional Network (TCN) is adopted as the temporal feature extraction backbone of the proposed model. Unlike recurrent neural networks, the TCN models sequential dependencies through stacked one-dimensional convolutions, enabling parallel feature extraction while preserving temporal causality [26]. In this study, the TCN mainly relies on dilated causal convolution and residual connections to capture multi-scale temporal dependencies in soil moisture and related environmental variables.

For a one-dimensional input sequence, the dilated causal convolution at time step *t* can be expressed as

(7) $$y_{t}=\sum_{k=0}^{K-1}w_{k}x_{t-rk},$$

where *K* denotes the convolution kernel size, *r* is the dilation factor, $w_{k}$ represents the convolution kernel weight, and $x_{t-rk}$ denotes the historical input. The causal structure ensures that the prediction at the current time step depends only on current and historical information, thereby avoiding the use of future information. Increasing the dilation factor enlarges the temporal receptive field without substantially increasing the computational cost, which is beneficial for modeling the lagged response of soil moisture to meteorological variations.

Residual connections are further employed in each TCN block to improve gradient propagation and training stability. The output of a residual block is expressed as

(8) $$H_{out}=\phi \left(F\left(H_{in}\right)+P\left(H_{in}\right)\right),$$

where F(·) denotes the transformation of the dilated causal convolution layers, P(·) denotes the projection mapping used for channel matching, and ϕ(·) denotes the activation function. The specific TCN architecture and parameter configuration used in the proposed MTC-TCN-Attn model are described in Section 3.5.3.

### 3.4. Self-Attention Mechanism and Multi-Task Learning

In soil moisture prediction tasks, to better capture the complex nonlinear relationships among multiple factors and to enhance the model’s predictive capability, this paper introduces the self-attention mechanism and multi-task learning (MTL) techniques to effectively model the temporal data of soil moisture. The following provides a detailed introduction to these two techniques:

#### 3.4.1. Self-Attention Mechanism

The self-attention mechanism computes the relationships between different parts of the input data to weight the importance of each time step, thereby dynamically adjusting the influence of different positions [28]. It has been widely used in deep learning tasks, particularly excelling in natural language processing and time-series data analysis. Unlike traditional convolutional or recurrent network architectures, self-attention can model sequence dependencies globally, but, as it inherently lacks positional information, it typically needs to be combined with positional encoding to capture the temporal order of the sequence.

Basic Idea of the Self-Attention Mechanism: The self-attention mechanism maps the input data into three distinct spaces: query (Q), key (K), and value (V). By computing the similarity between the query and the key, a weight matrix is obtained, which is then used to weight the values, producing the weighted output. This mechanism dynamically adjusts the importance of different time steps and captures crucial temporal features.

Self-Attention Mechanism Formula: Given an input sequence $X=\{x_{1},x_{2},\ldots ,x_{T}\}$, where *T* denotes the sequence length, the query (*Q*), key (*K*), and value (*V*) matrices are obtained via distinct linear transformations applied to the input sequence. The output of the self-attention mechanism is computed as

(9) $$\mathrm{Attention}\left(Q,K,V\right)=\mathrm{softmax}\left(\frac{QK^{T}}{\sqrt{d_{k}}}\right)V$$

where *Q*, *K*, and *V* are the query, key, and value matrices, respectively; $d_{k}$ is the dimension of the key vectors; and the term $\frac{QK^{T}}{\sqrt{d_{k}}}$ computes the similarity between queries and keys (scaled by $\frac{1}{\sqrt{d_{k}}}$ to avoid excessively large values). The softmax function then normalizes these similarity scores to obtain the weight coefficients for each value vector.

Through this mechanism, self-attention captures global information from the input sequence and dynamically assigns weights to key time steps. Compared with traditional convolutional or recurrent neural networks, self-attention achieves superior performance in modeling long-range dependencies in sequential data.

#### 3.4.2. Multi-Task Learning

Multi-task learning (MTL) is a machine learning approach that trains multiple tasks simultaneously within a single model to improve generalization [23]. Each task shares part of the network architecture while having its own output layer, and overall prediction performance is enhanced through the sharing of low-level features across tasks.

In soil moisture prediction, different soil-moisture-related variables exhibit complex interdependencies. To simultaneously predict multiple soil-moisture-related targets, this paper adopts a multi-task learning framework. By sharing the feature extraction network, the framework ensures that the model can handle multiple prediction tasks concurrently, avoiding the overfitting issues commonly encountered in traditional single-task learning methods and improving prediction accuracy.

Note: A multi-task learning framework is also called a multi-task collaborative framework.

Advantages of multi-task learning:

1. Knowledge Sharing: Tasks share partial network structures, which helps reduce the sample requirement for each task and improve training efficiency.
2. Mitigating Overfitting: By learning shared features across multiple tasks, multi-task learning enhances the model’s generalization ability and reduces overfitting in individual tasks.
3. Improved Prediction Accuracy: By sharing information across multiple tasks, the model can learn richer features, thereby improving prediction performance for each task.

In this paper, soil moisture prediction is formulated as a multi-task regression problem, where each task predicts a soil-moisture-related variable, such as surface soil moisture, deep soil moisture, and soil moisture change rate. Through the multi-task learning framework, the model can learn features for multiple tasks simultaneously, thereby improving the accuracy of soil moisture prediction.

### 3.5. MTC-TCN-Attn: The TCN-Attention-Based Multi-Task Collaborative Prediction Method for Wheat Soil Moisture

**Figure 3:**
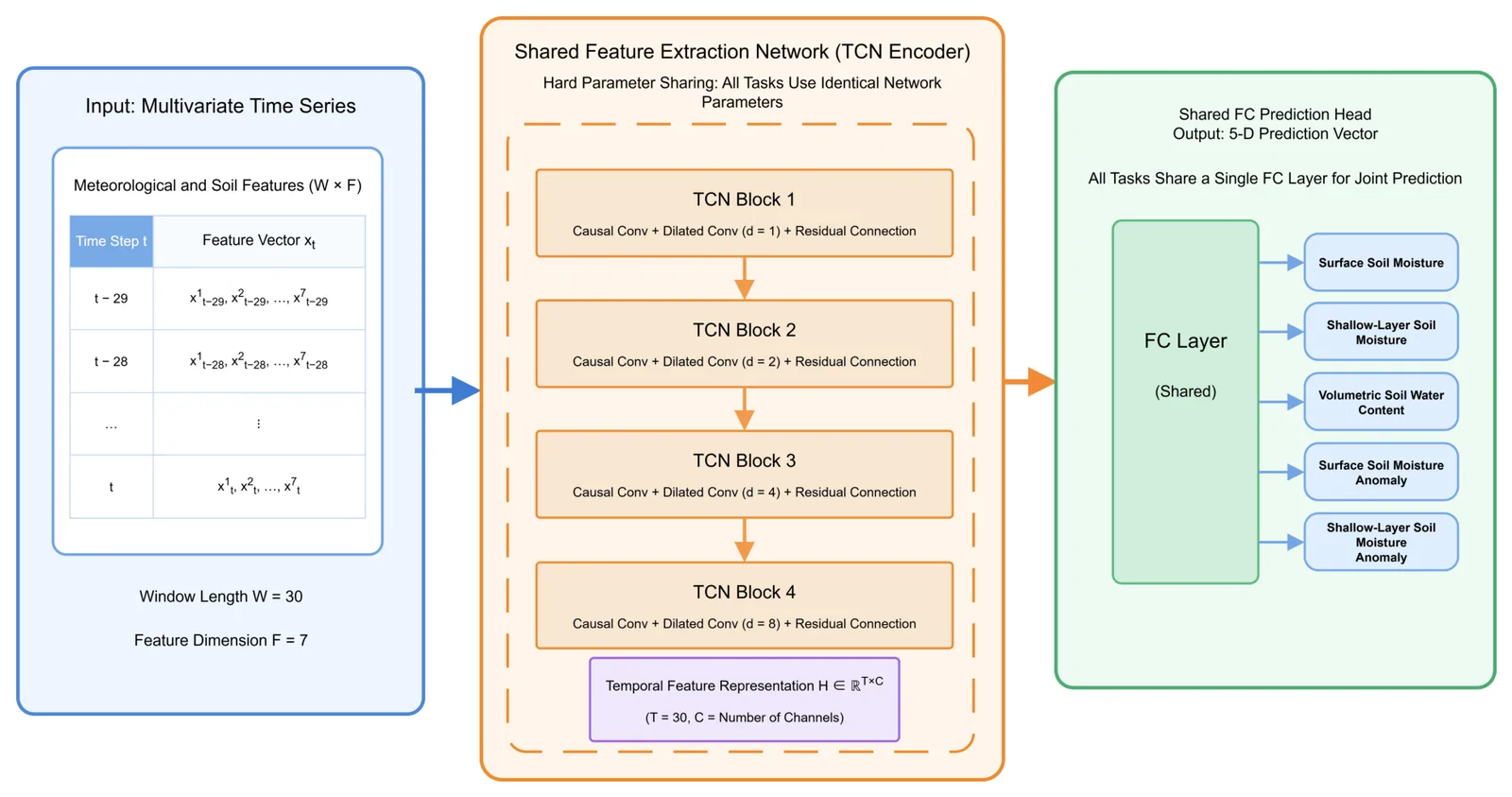
The MTC-TCN-Attn model.

In this paper, a multi-task learning model based on a Temporal Convolutional Network (TCN) and an attention mechanism, named the MTC-TCN-Attn model, is proposed. The MTC-TCN-Attn model employs the TCN to efficiently model time series while incorporating an attention mechanism to dynamically weight the input features of multiple variables, thereby highlighting key influencing factors. Building on this, a multi-task learning framework is constructed to achieve the joint prediction of soil moisture at different soil layers and related environmental variables. This framework fully exploits the coupling relationships and information complementarity among distinct variables and soil layers, improving the overall prediction accuracy and stability of the model. This approach offers a new perspective for the refined prediction of multi-layer soil moisture in complex environmental systems and holds significant application value for intelligent agricultural management and the optimal allocation of water resources. The overall flowchart of this study is presented in Figure 3.

#### 3.5.1. Sample Splitting and Sliding Window Construction

To convert the time series into supervised learning samples, a sliding window is adopted to construct input sequences. Let the window length be *W* and the prediction horizon be *H*. In this study, W=30 and H=1 are set, meaning the 5 target variables at the next time step are predicted using the input sequence from the past 30 days.

For any time step *t*, the input sample is expressed as

(10) $$X_{t}=\left[x_{t-W+1},x_{t-W+2},\ldots ,x_{t}\right]\in R^{W\times F}$$

where F=7 is the feature dimension. The corresponding output is

(11) $$Y_{t}=y_{t+H}\in R^{K}$$

where K=5 is the number of tasks.

The total number of samples is calculated as

(12) N=T−W−(H−1)

where *T* is the total length of the time series.

For train/test set split, the data is divided chronologically with a training set ratio of 0.8 to avoid information leakage. The number of training samples is set as

(13) $$N_{\mathrm{train}}=\left\lfloor 0.8N\right\rfloor ,\;N_{\mathrm{test}}=N-N_{\mathrm{train}}$$

This ensures that the model always faces unseen future data during the test phase, which is consistent with the actual prediction scenario.

This study adopts a chronological holdout validation strategy rather than random cross-validation. All supervised learning samples are arranged in chronological order, with the first 80% used for model training and the remaining 20% used as an independent test set. This ensures that all test samples occur later in time than the training samples, thereby better reflecting the practical scenario of forecasting future time steps. Furthermore, to examine whether the model performance depends on a single 80%/20% split, two additional year-based temporal holdout experiments are conducted. Specifically, data from 2016–2019 are used for training and data from 2020 for testing, while data from 2017–2020 are used for training and data from 2021 for testing.

#### 3.5.2. Multi-Task Learning Framework and Baseline Model Setup

1. Multi-Task Learning Framework Design
  This study adopts a hard parameter sharing strategy to construct the multi-task learning framework. The core idea of this strategy is that multiple prediction tasks share the same underlying feature extraction network, with separation occurring only at the output layer, thereby enabling cross-task sharing of feature representations and knowledge transfer. Specifically, the TCN encoder serves as the shared backbone network for all tasks, extracting a unified temporal feature representation. A shared fully connected regression head then outputs five predictions, corresponding to the five target variables: SSM, SUSM, SMP, SSMA, and SUSMA.
  Compared with soft parameter sharing, the hard parameter sharing structure offers advantages such as fewer parameters, more stable training, and a stronger regularization effect. In the meteorological and soil moisture datasets used in this study, the five target variables are intrinsically related through shared hydrological and statistical characteristics. Specifically, SSM and SUSM represent soil water storage in the surface and subsurface/root-zone model layers, respectively, while SMP reflects the overall moisture status of the soil profile. In addition, SSMA and SUSMA characterize the anomaly states corresponding to SSM and SUSM. These relationships provide a physical and statistical basis for joint representation learning within the multi-task framework. Hard parameter sharing can explicitly exploit these correlations among multiple variables, reducing the risk of overfitting in single-task, small-sample scenarios and enhancing the overall generalization ability of the model.
2. Baseline Model Setup
  To systematically validate the effectiveness of each module, this study establishes three categories of comparative models, detailed as follows:
  - Model M1: Single-Task TCN. Five independent TCN models are trained separately for the five target variables. All models use the same TCN architecture and hyperparameter settings, and differ only in their prediction targets. Each model is independently initialized and trained for its corresponding target, without parameter sharing among tasks. This setup serves as a baseline reference without multi-task learning and is used to evaluate the effect of the multi-task sharing strategy.
  - Model M2: Multi-Task TCN. This model employs a hard parameter sharing multi-task TCN structure, where the shared encoder outputs five-dimensional predictions without the incorporation of any attention mechanism. It serves as the baseline for the multi-task architecture, used to evaluate the effect of multi-task joint training itself, and forms an ablation comparison with the main model.
  - Model M3: Multi-Task TCN + Self-Attention (MTC-TCN-Attn). Building upon the Multi-Task TCN, a multi-head self-attention (MHA) module is introduced after the TCN encoder, with a residual connection structure employed to prevent information degradation. By computing global dependencies among all time steps within the sequence, the self-attention module adaptively weights critical time steps, thereby compensating for the limitations of convolutional structures in temporal weight allocation.
3. Unique Differences Between Models and Interpretability of Comparison
  To ensure a fair comparison, the three models adopt the same basic TCN configuration and training input settings. Specifically, the sliding-window length is set to W=30, and four TCN residual blocks are employed. Each TCN block contains 64 channels with a kernel size of 3, while the dilation rates of the four blocks are set to {1,2,4,8}, respectively. The dropout rate is fixed at 0.2 for all models. For the proposed M3 model, the self-attention module contains four attention heads with a key dimension of 16, whereas no attention mechanism is incorporated into M1 or M2.
  The key architectural and training differences among the three comparative models are summarized in Table 2. M1 consists of five independently trained single-task TCN models, with each model predicting one target variable. M2 adopts a hard parameter-sharing strategy and jointly predicts the five target variables using a single shared TCN model. M3 retains the same multi-task structure as M2 while introducing a multi-head self-attention module for adaptive temporal feature weighting. In terms of model complexity, M1 contains 105,089 trainable parameters per model and 525,445 parameters in total for the five independently trained models, whereas M2 contains 105,349 trainable parameters and M3 contains 109,685 trainable parameters.

The comparison between M1 and M2 verifies the effectiveness of the multi-task learning framework itself; the comparison between M2 and M3 validates the independent contribution of the self-attention module; and the comparison between M1 and M3 comprehensively evaluates the overall improvement of the proposed method relative to the simplest baseline. These three control relationships form a complete ablation study chain, ensuring the interpretability and persuasiveness of the conclusions.

#### 3.5.3. Temporal Feature Modeling Method Based on Temporal Convolutional Network

To effectively characterize the dynamic variations and long-term dependencies of soil moisture at different soil layers and related environmental factors, this study introduces the TCN as the core temporal feature modeling module in the multi-task prediction model. Compared with traditional recurrent neural networks (RNNs), the TCN, through its causal and dilated convolutional structures, significantly expands the receptive field while ensuring temporal causality. This enables the model to more efficiently capture the lag effects and multi-scale temporal features arising from the influence of factors such as precipitation and temperature on soil moisture. Therefore, the TCN is employed to construct a unified temporal feature representation, providing high-quality feature inputs for the subsequent multi-task prediction. Based on the introduction to the fundamental principles of the TCN in Section 3.3 (Temporal Convolutional Network), this section focuses on elaborating the input representation, structural configuration, and specific implementation of the TCN module in the proposed model.

1. Input Representation and Symbol Definitions
  Let the length of the input sequence window be denoted by *W*, and the number of features be denoted by *F*. For the *n*-th sample, the input sequence can be expressed as
  (14) $$X^{\left(n\right)}=\left[x_{1}^{\left(n\right)},x_{2}^{\left(n\right)},\ldots ,x_{W}^{\left(n\right)}\right]\in R^{W\times F},$$
  where $x_{t}^{\left(n\right)}\in R^{F}$ denotes the multi-variate feature vector at the *t*-th time step within the window. In this paper, the window length is set to W=30, and the input feature dimension is set to F=7. In the TCN, the hidden representation of the *l*-th layer is denoted as
  (15) $$H^{\left(l\right)}\in R^{W\times C_{l}},$$
  where $C_{l}$ denotes the number of channels in the *l*-th layer. In this paper, it is uniformly set to $C_{l}=64$, and $H^{\left(0\right)}=X$.
2. Multi-channel Dilated Causal Convolution Modeling
  To ensure the causality of time-series modeling and effectively capture long-term dependencies, a dilated causal convolution structure is adopted in the TCN module. During the convolution operation, this structure uses only the information from the current and historical time steps, thereby preventing future information leakage.
  Meanwhile, by introducing dilation factors into the convolution operation, the model can enlarge the temporal receptive field without increasing computational complexity, thereby effectively modeling the lag effects of soil moisture caused by processes such as precipitation and evapotranspiration.
  For multi-channel input features, the TCN employs multiple convolution kernels at each layer to fuse information from different channels, enabling joint modeling of multi-variate time-series features. As the network depth increases and the dilation factor grows layer by layer, the model can progressively capture multi-scale temporal dependencies ranging from short-term variations to long-term trends.
3. Residual Connection and Channel Matching
  To improve the training stability of deep networks and alleviate the gradient vanishing problem, a residual connection is introduced into each TCN block. Let the input feature be denoted by $H_{\mathrm{in}}$, and let the main branch transformation be denoted by F(·). The output of the block is given by
  (16) $$H_{\mathrm{out}}=\phi \left(F\left(H_{\mathrm{in}}\right)+P\left(H_{\mathrm{in}}\right)\right),$$
  where P(·) denotes the projection mapping, which is used for dimensional matching when the number of channels is inconsistent. In this paper, a 1×1 convolution is employed to implement the projection mapping, ensuring that the residual connection can be performed properly. The activation function ϕ(·) is implemented using the ReLU function.
4. Specific Configuration of the TCN Module
  Considering the data scale and the complexity of the prediction task, the TCN module in this study is configured as follows: the number of blocks is set to B=4; each block contains two convolutional layers; the convolution kernel size is set to k=3; the number of output channels is set to 64; the dilation factor is shared by the two convolutional layers within each block, and the dilation factors of the four blocks are set to {1,2,4,8} in sequence; and the dropout rate is set to 0.2.
  The deep TCN effectively enlarges the temporal receptive field through causal convolution and dilated convolution, and the residual structure improves training stability. However, its essence remains a local pattern extraction method based on convolution kernels. The weight allocation of convolution operations along the temporal dimension is relatively fixed; that is, the dependency relationships among different time steps are implicitly determined by the convolution kernel parameters, and the model lacks the ability to dynamically assign weights to key time points.
  In the process of soil moisture prediction, precipitation events, evapotranspiration processes, and temperature variations often exhibit nonuniform lag effects. Different historical time periods may contribute significantly differently to the current prediction result. Therefore, relying solely on the convolutional structure may make it difficult to fully capture global temporal dependencies.
  Based on the above considerations, a self-attention mechanism is introduced after the TCN feature extraction module to achieve dynamic weighting and global modeling of temporal information, thereby further enhancing the model’s ability to represent complex temporal dependencies.

#### 3.5.4. Temporal Dependency Modeling Method Based on Self-Attention Mechanism

To further characterize the global dependencies within the time series and the differential impacts of different time steps on the prediction results, and to compensate for the limitations of convolutional structures in temporal weight allocation, this study introduces a multi-head self-attention mechanism after the TCN shared feature extraction module, establishing a temporal dependency modeling method based on the self-attention mechanism. This method can adaptively learn the correlation strengths among various time steps within the global temporal range and dynamically weight key time nodes, thereby enhancing the model’s capability to represent complex temporal dependencies.

Compared with convolutional operations that extract features based on a fixed receptive field, the self-attention mechanism can establish global dependencies across the entire time window and adaptively assign importance weights to different time steps according to the input data, thereby more effectively capturing the nonuniform lag effects exerted on soil moisture by factors such as precipitation, evapotranspiration, and temperature.

1. Input Feature Representation
  Let the temporal feature representation extracted by the TCN be denoted as
  (17) $$H\in R^{T\times C},$$
  where *T* denotes the sequence length, and *C* denotes the number of channels or the feature dimension. Moreover, $H={\left[h_{1},h_{2},\ldots ,h_{T}\right]}^{T}$, where $h_{t}\in R^{C}$ denotes the high-level feature vector at the *t*-th time step.
2. Self-Attention-Based Feature Modeling
  In the self-attention module, the input features are first mapped into the query, key, and value spaces through linear transformations, as shown in
  (18) $$Q=HW_{Q},\;K=HW_{K},\;V=HW_{V},$$
  where $W_{Q},W_{K},W_{V}\in R^{C\times d_{k}}$ are trainable parameter matrices, and $d_{k}$ denotes the feature mapping dimension.
  The relevance weights among different time steps are then calculated as
  (19) $$A=\frac{QK^{T}}{\sqrt{d_{k}}},$$
  and the attention weight matrix is obtained by normalization through the softmax function:
  (20) α=softmax(A).
  The final output is given by
  (21) Z=αV.
  This process realizes the weighted aggregation of the entire time-series information, enabling the model to dynamically learn the contribution of different historical time steps to the current prediction target.
3. Multi-Head Attention Mechanism
  The computation process of multi-head self-attention is shown in
  (22) $${\mathrm{head}}_{i}=\mathrm{Attention}\left(Q_{i},K_{i},V_{i}\right),$$
  where each attention head independently learns temporal dependency relationships in different feature subspaces. The outputs of all heads are concatenated and then transformed through a linear mapping to obtain the final output:
  (23) $$\mathrm{MHA}\left(H\right)=\mathrm{Concat}\left({\mathrm{head}}_{1},\ldots ,{\mathrm{head}}_{h}\right)W_{O},$$
  where $W_{O}$ denotes the output projection matrix. The multi-head mechanism enables the model to simultaneously focus on dependency relationships at different temporal scales, thereby enhancing its representation capability for complex time-series features.
4. Residual Connection and Layer Normalization
  To improve training stability, residual connection and layer normalization are introduced into the attention module. The computation is formulated as
  (24) $$H^{\prime }=H+\mathrm{Dropout}\left(\mathrm{MHA}(\mathrm{LayerNorm}(H))\right),$$
  where LayerNorm is used to stabilize the feature distribution, Dropout is used to suppress overfitting, and the residual connection is used to ensure stable information propagation.
5. Module Function Analysis
  The TCN module can effectively extract local temporal patterns and multi-scale temporal dependencies. However, its importance allocation for different time steps is implicit and fixed, making it difficult to characterize the dynamic influence of key temporal nodes.
  By introducing the self-attention mechanism, the model can adaptively learn the contribution weights of each time step over the global temporal range, thereby more accurately identifying the influence of key driving factors, such as precipitation events and evapotranspiration processes, on soil moisture variations. This mechanism complements the TCN and effectively improves the model’s ability to capture complex temporal dependencies and its prediction accuracy.
6. Module Parameter Configuration
  Considering the experimental requirements and model complexity control, the self-attention module in this study is configured as follows: the number of attention heads is set to 4, the key vector dimension is set to 16, the dropout rate is set to 0.2, and the module is placed after the TCN output and before the pooling layer.

#### 3.5.5. Multi-Task Joint Loss Function and Optimization Strategy

Because all output targets were standardized using Z-score normalization, differences in numerical scale across tasks were substantially reduced. Accordingly, an equally weighted joint loss function was adopted to optimize the multi-task model. The overall loss function is defined as

(25) $$L=\sum_{k=1}^{K}w_{k}L_{k},$$

where $L_{k}$ denotes the loss function of the *k*-th task, and $w_{k}$ represents the corresponding task weight. Equal weights were assigned to all tasks as follows:

(26) $$w_{1}=w_{2}=\cdots =w_{K}=\frac{1}{K}.$$

This strategy enables the joint learning of multiple soil-moisture-related variables while maintaining stable model training.

It should be noted that the gradient directions of different tasks may be inconsistent during multi-task learning, potentially leading to negative transfer between tasks. This issue is particularly relevant to statistical variables such as SSMA and SUSMA, whose variation patterns differ to some extent from those of the original soil moisture variables. Consequently, these variables may be adversely affected by other tasks during shared-parameter updates.

To alleviate these problems, data standardization, residual connections, dropout, self-attention, and gradient clipping were employed to improve training stability and strengthen the model’s ability to represent key temporal features. The experimental results indicate that incorporating the attention mechanism markedly improves the overall predictive performance of the multi-task model.

## 4. Results

The experiments are organized to evaluate the proposed MTC-TCN-Attn framework from four complementary perspectives. First, the internal comparison among the Single-Task TCN, Multi-Task TCN, and MTC-TCN-Attn examines whether multi-task parameter sharing alone is sufficient and whether self-attention provides additional performance gains. Second, comparisons with SVR, LSTM, Transformer, and MT-LSTM evaluate whether the superiority of the proposed model extends beyond the TCN-based comparison framework. Third, temporal robustness experiments investigate whether the observed performance remains stable when different chronological periods are used for training and testing. Finally, gradient conflict analysis examines whether heterogeneous optimization interactions among the five tasks are associated with the nonuniform performance gains observed in multi-task learning. Unless otherwise stated, model performance is evaluated using the coefficient of determination ($R^{2}$), root mean square error (RMSE), and mean absolute error (MAE).

### 4.1. Internal Comparison: Effects of Multi-Task Sharing and Self-Attention

The internal comparison reveals a clear two-stage effect of the proposed framework. Introducing hard parameter sharing alone does not consistently improve the principal soil-moisture targets; however, incorporating self-attention after the shared TCN encoder substantially changes this outcome. The complete MTC-TCN-Attn model achieves the best performance for SSM, SUSM, and SMP among the three TCN-based models, indicating that effective multi-task prediction requires not only shared representations but also an enhanced temporal representation mechanism.

The quantitative results for the five targets are reported in Table 3, Table 4 and Table 5.

A direct comparison between the Single-Task TCN and Multi-Task TCN shows that hard parameter sharing alone does not produce uniform improvements. For SSM, SUSM, and SMP, the $R^{2}$ values decrease from 0.8882, 0.9614, and 0.9583 to 0.8645, 0.9465, and 0.9431, respectively. In contrast, SSMA improves markedly from 0.4905 to 0.6216, while SUSMA remains almost unchanged. Thus, simple multi-task sharing produces clearly task-dependent effects rather than a uniform performance gain.

The introduction of self-attention substantially improves the performance of the three principal soil-moisture targets. Compared with the Multi-Task TCN, MTC-TCN-Attn increases the $R^{2}$ values of SSM, SUSM, and SMP by 0.0502, 0.0186, and 0.0191, respectively. More importantly, the resulting performance not only recovers the degradation caused by simple parameter sharing but also exceeds that of the corresponding Single-Task TCN models. In terms of RMSE, self-attention reduces the errors of the Multi-Task TCN by approximately 20.7%, 19.3%, and 18.3% for SSM, SUSM, and SMP, respectively.

**Figure 4:**
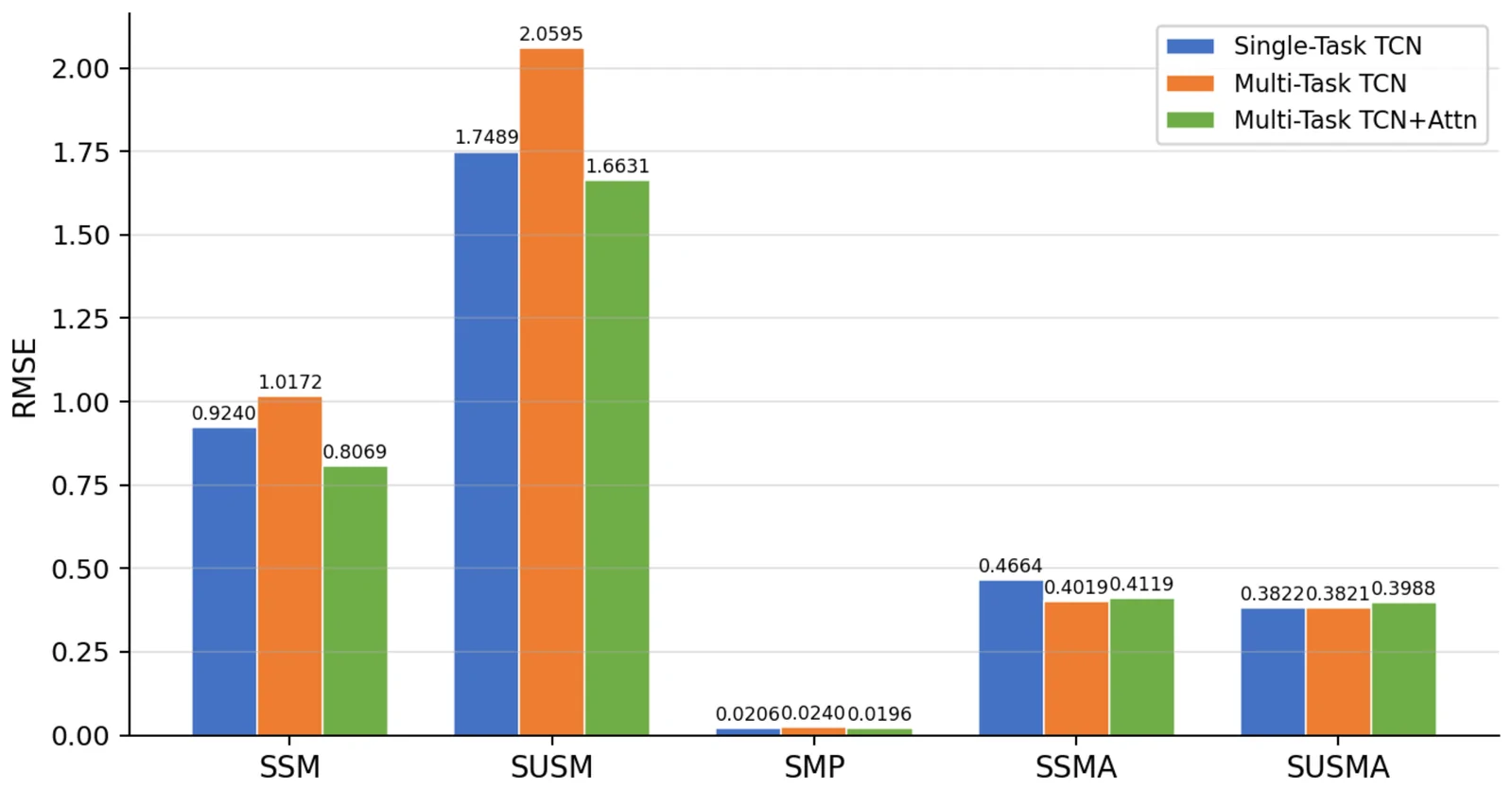
RMSE comparison of the three TCN-based models across five prediction targets.

**Figure 5:**
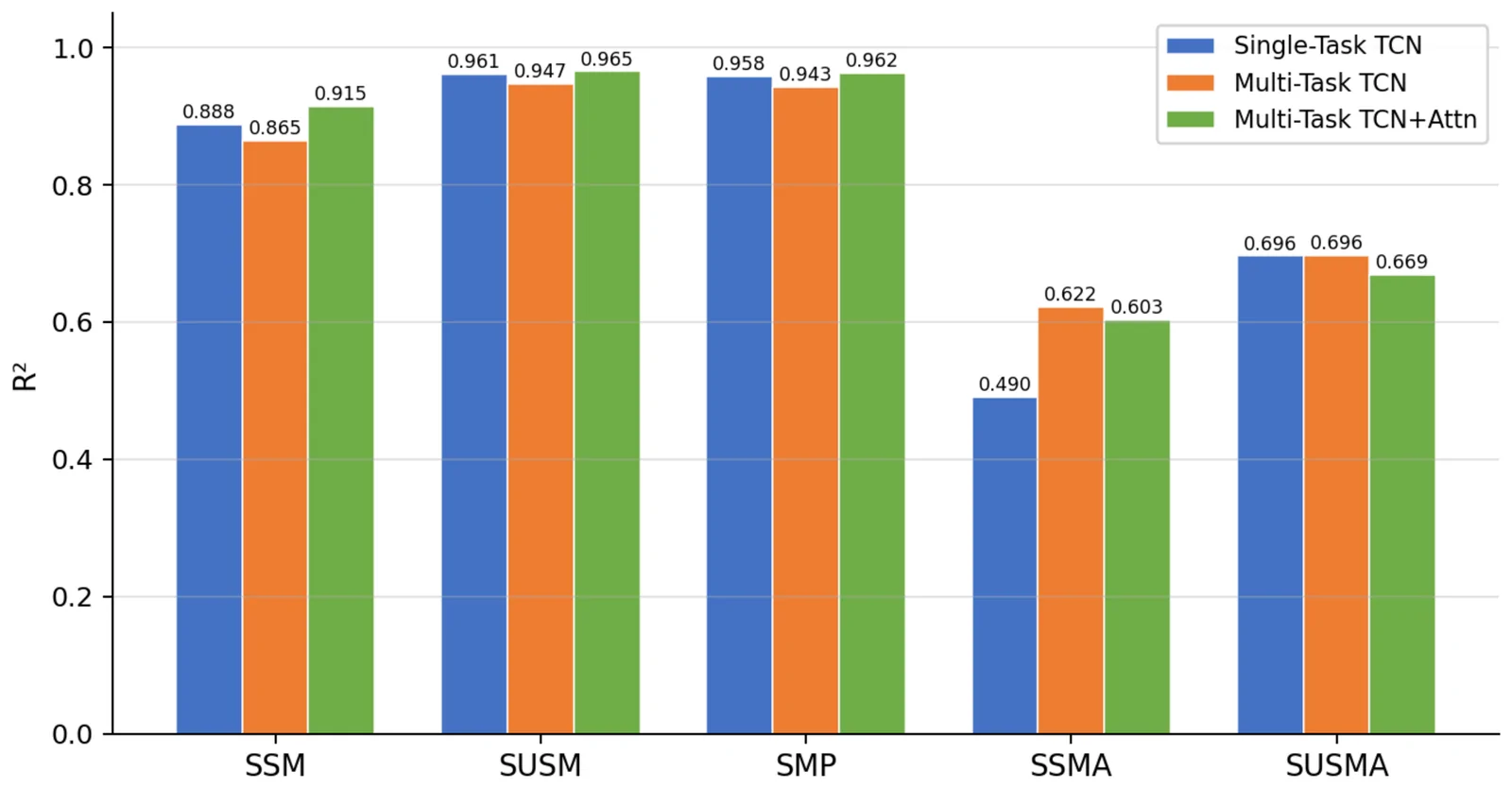
$R^{2}$ comparison of the three TCN-based models across five prediction targets.

Figure 4 and Figure 5 provide a visual summary of this performance pattern. The advantage of MTC-TCN-Attn is particularly evident for the three principal targets, whereas the anomaly-based targets exhibit a different response to the multi-task architecture.

**Figure 6:**
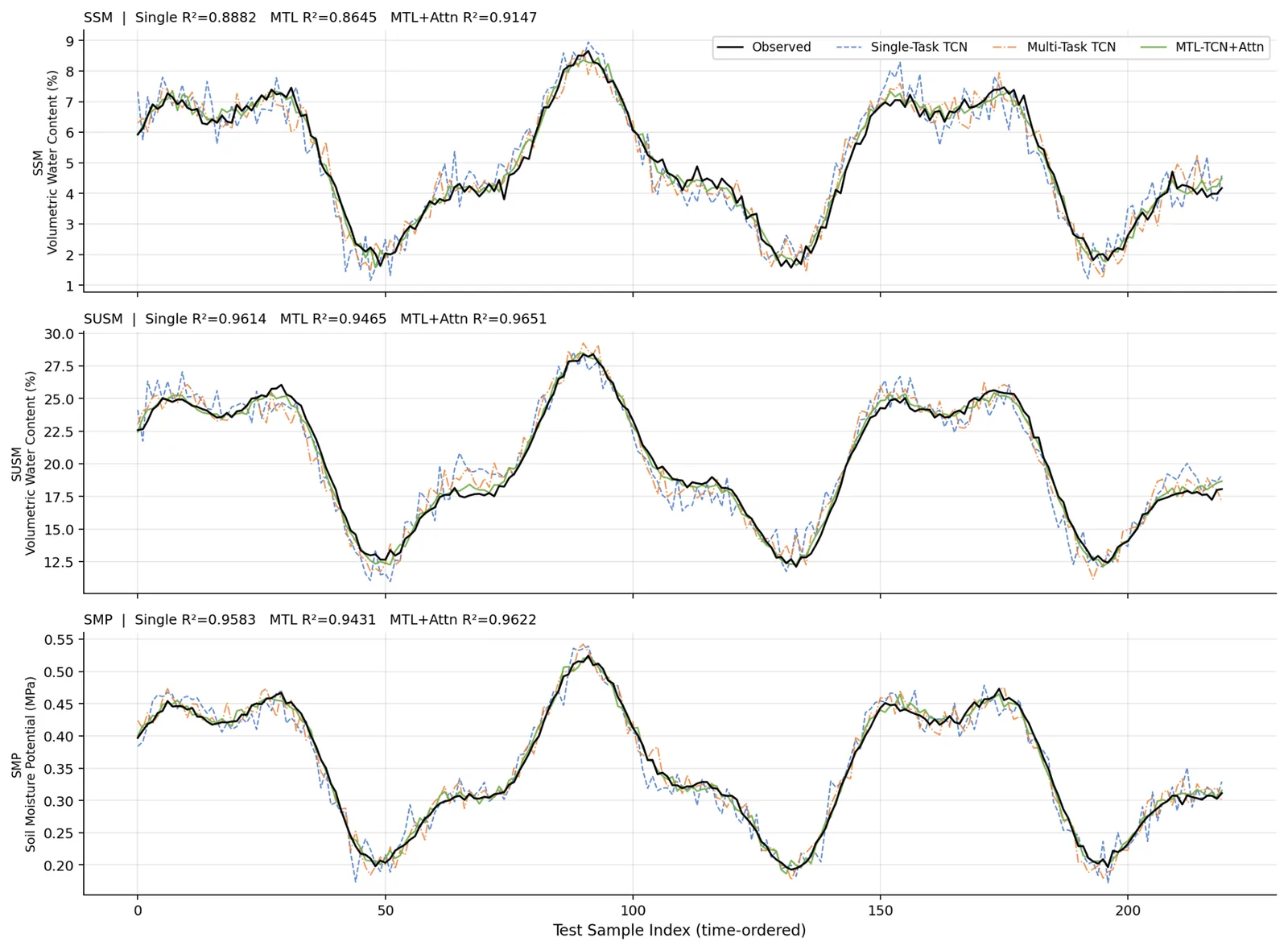
Time-series prediction comparison of the three TCN-based models for SSM, SUSM, and SMP.

The time-series results in Figure 6 further confirm the strong predictive capability of MTC-TCN-Attn for SSM, SUSM, and SMP. Its predicted trajectories closely follow the major temporal variations of the observed series, including rising and falling phases and local extrema.

**Figure 7:**
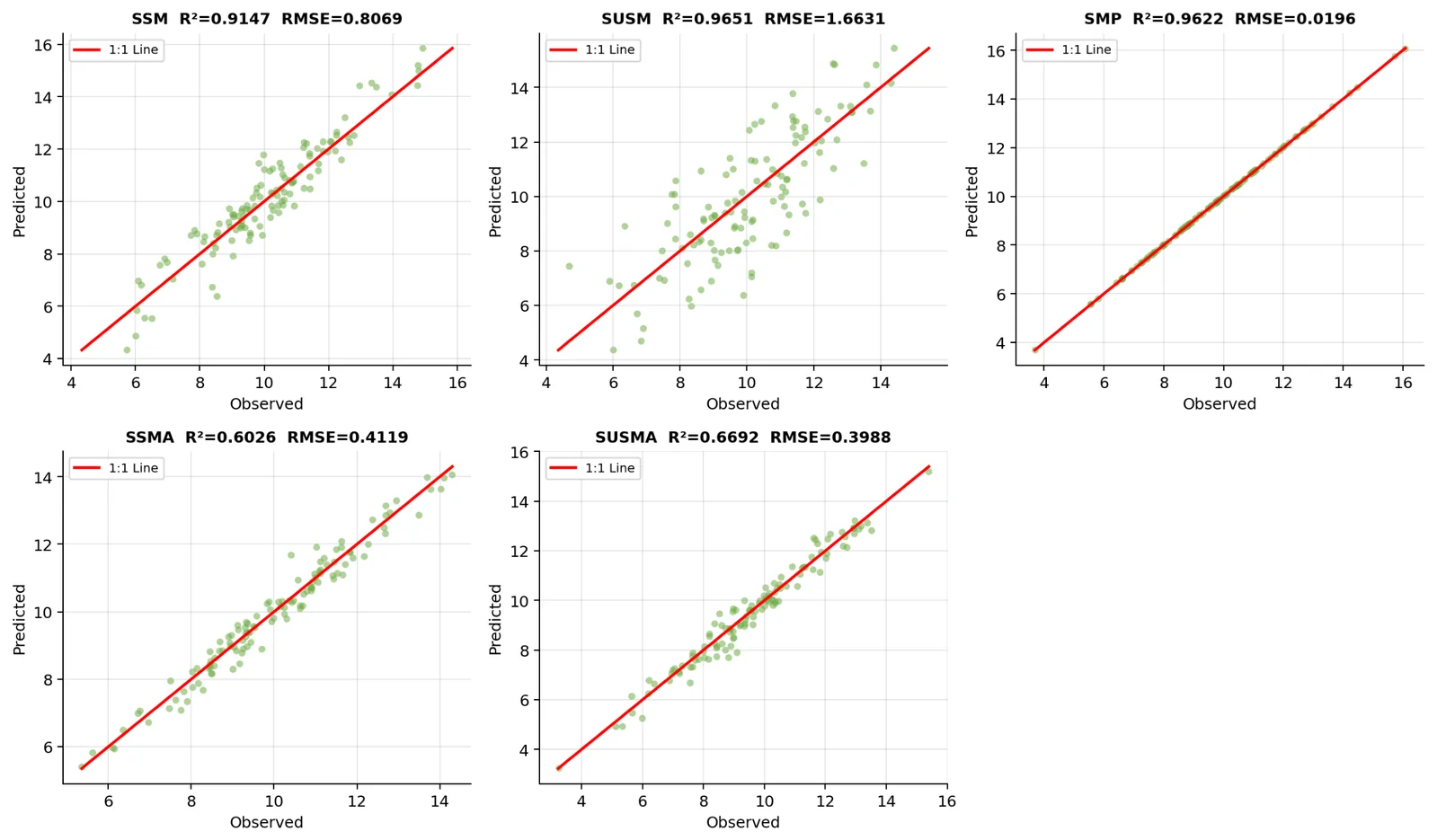
Scatter plots of observed and predicted values obtained by MTC-TCN-Attn for the five prediction targets.The green dots represent individual observed–predicted data pairs, while the red lines indicate the 1:1 reference lines.

A similar pattern is observed in Figure 7. Predictions for SSM, SUSM, and SMP are concentrated close to the 1:1 reference line, consistent with their high $R^{2}$ and low error values, while the anomaly-based variables exhibit greater dispersion.

**Figure 8:**
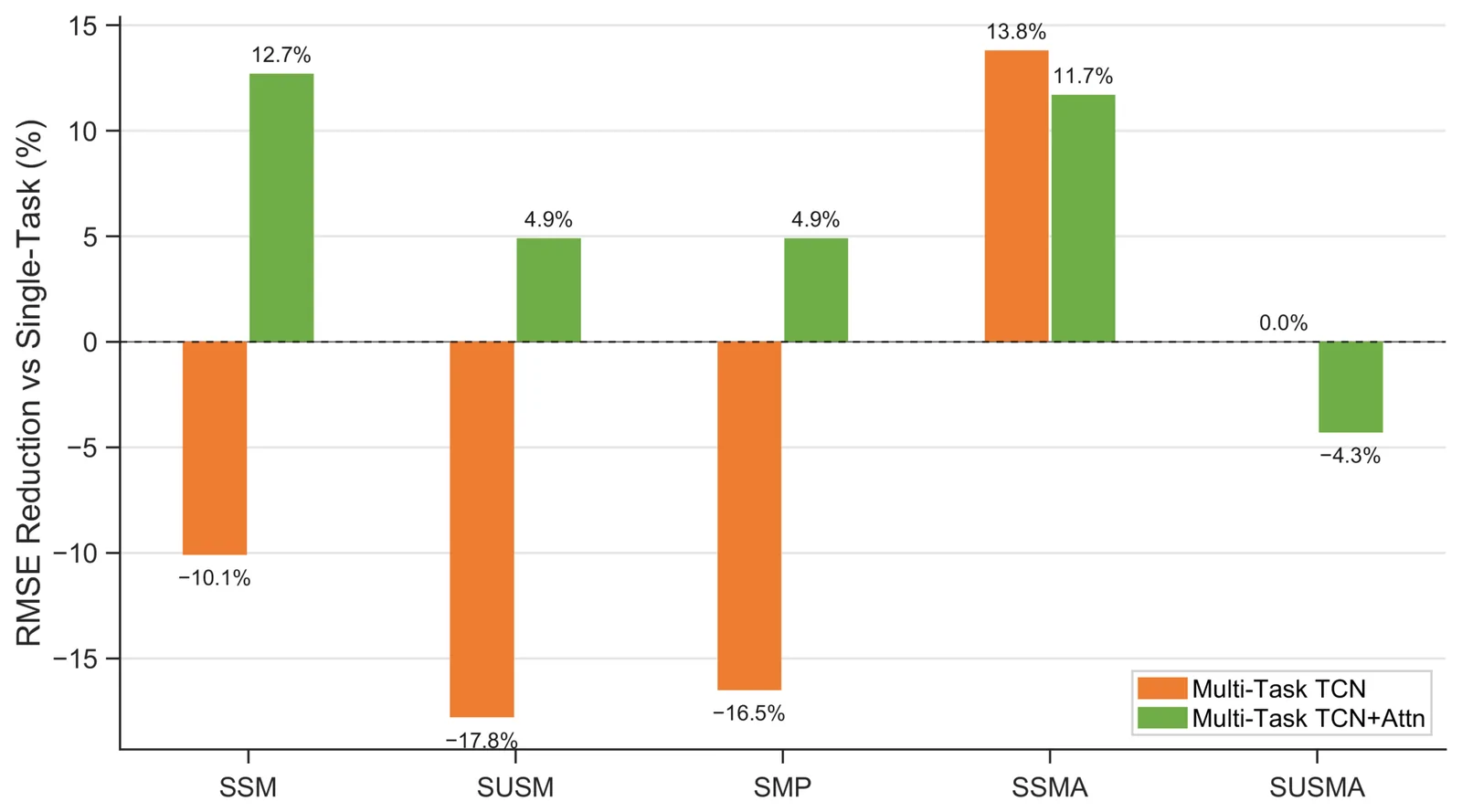
RMSE reduction rate (%) of the multi-task models relative to the Single-Task TCN baseline.

The overall gain relative to independent single-task prediction is summarized in Figure 8. Compared with the Single-Task TCN, the complete MTC-TCN-Attn model reduces RMSE by approximately 12.7%, 4.9%, and 4.9% for SSM, SUSM, and SMP, respectively. SSMA also shows an RMSE reduction of approximately 11.7%, whereas SUSMA increases by approximately 4.3%. Therefore, the internal comparison demonstrates that the principal advantage of the proposed architecture is concentrated on SSM, SUSM, and SMP, while the benefits are not completely uniform across all five tasks.

### 4.2. Comparison with Representative Benchmark Models

The benchmark experiment further demonstrates that the advantage of MTC-TCN-Attn is not restricted to comparisons within the TCN family. The proposed model is compared with four representative approaches covering different prediction paradigms: conventional nonlinear regression (SVR), recurrent sequence modeling (LSTM), attention-based sequence modeling (Transformer), and multi-task recurrent learning (MT-LSTM). This comparison therefore provides a broader assessment of whether the proposed combination of TCN, self-attention, and multi-task learning offers advantages over alternative modeling strategies.

The quantitative results are reported in Table 6, Table 7 and Table 8.

A consistent advantage is observed across all three evaluation metrics. MTC-TCN-Attn achieves the highest $R^{2}$ and the lowest RMSE and MAE for all five targets. This result is particularly important because no single external baseline dominates across all variables: LSTM performs strongly for SUSM, whereas Transformer provides the strongest external results for several other targets. In contrast, MTC-TCN-Attn maintains the best overall performance across the complete set of prediction tasks.

Relative to the strongest external benchmark for each individual target, MTC-TCN-Attn reduces RMSE by approximately 47.3%, 20.5%, 39.4%, 17.8%, and 12.6% for SSM, SUSM, SMP, SSMA, and SUSMA, respectively. The improvement is therefore not limited to the three principal targets but also extends to the two anomaly-based variables.

The comparison with MT-LSTM is particularly informative. Both MT-LSTM and MTC-TCN-Attn perform joint multi-output prediction, yet the proposed model achieves higher $R^{2}$ and lower RMSE and MAE for all five targets. This provides evidence that the performance gain is not explained by multi-task learning alone; the temporal representation architecture used by MTC-TCN-Attn contributes additional predictive capability.

**Figure 9:**
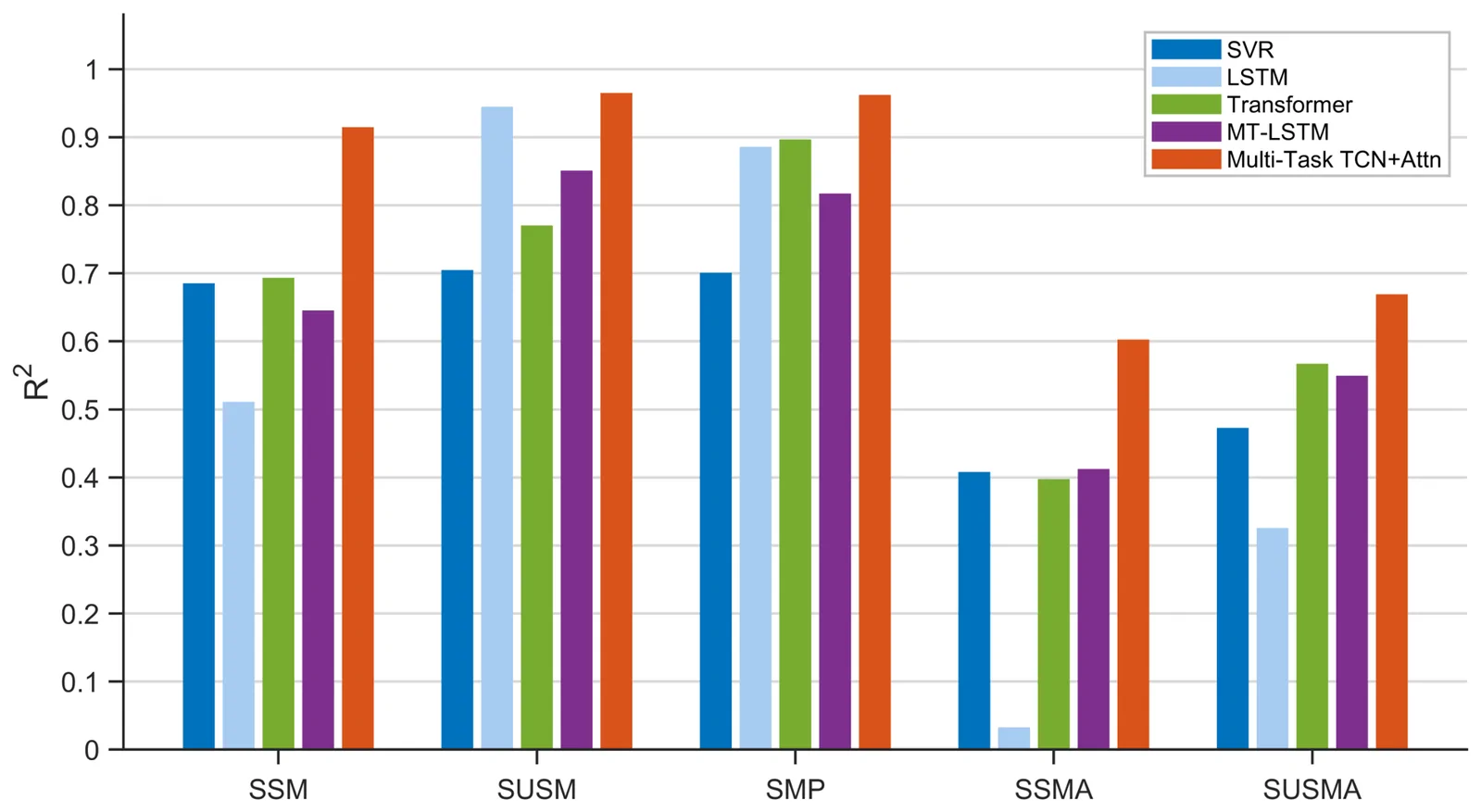
$R^{2}$ comparison of MTC-TCN-Attn with representative benchmark models across five prediction targets.

**Figure 10:**
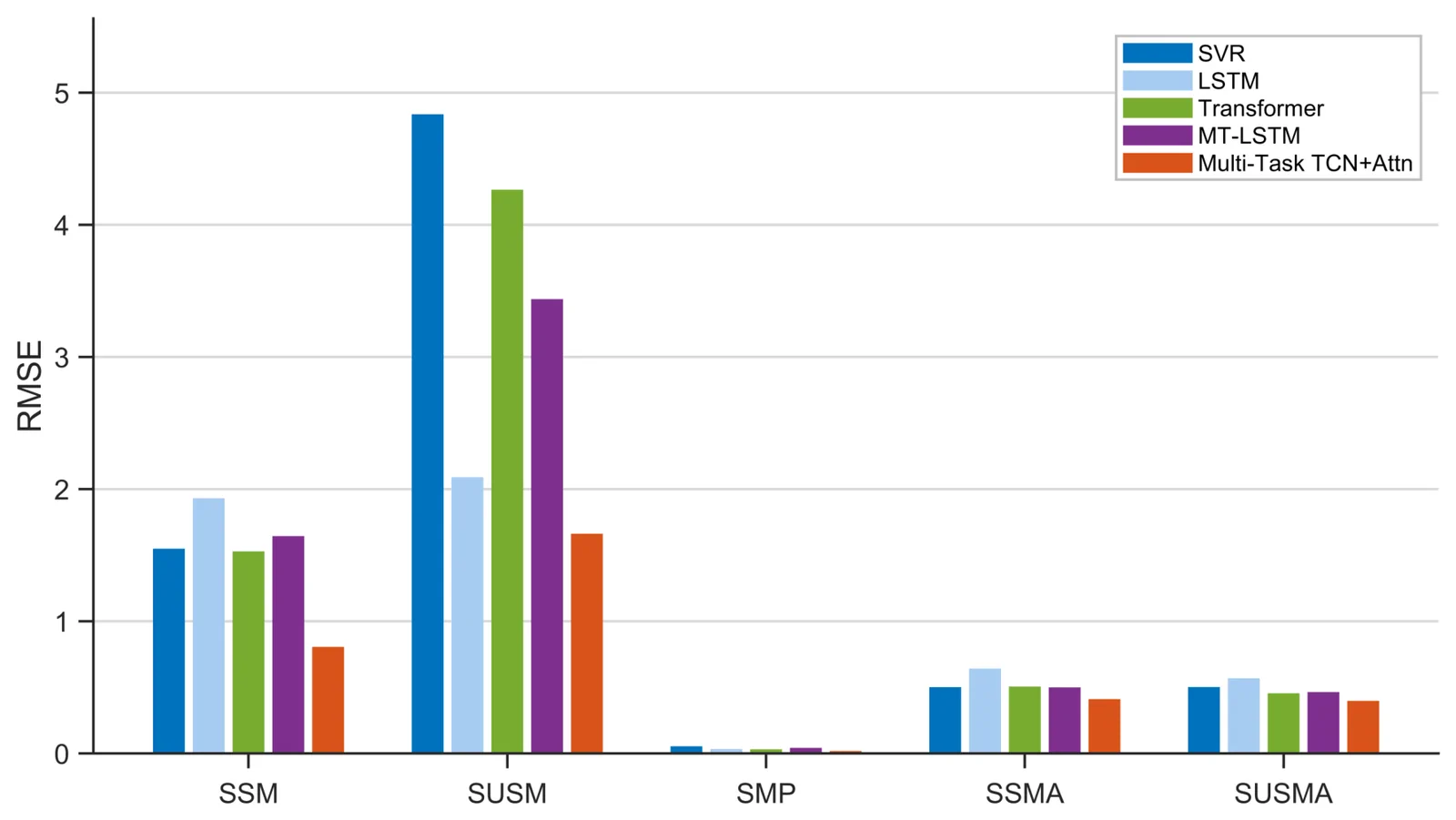
RMSE comparison of MTC-TCN-Attn with representative benchmark models across five prediction targets.

**Figure 11:**
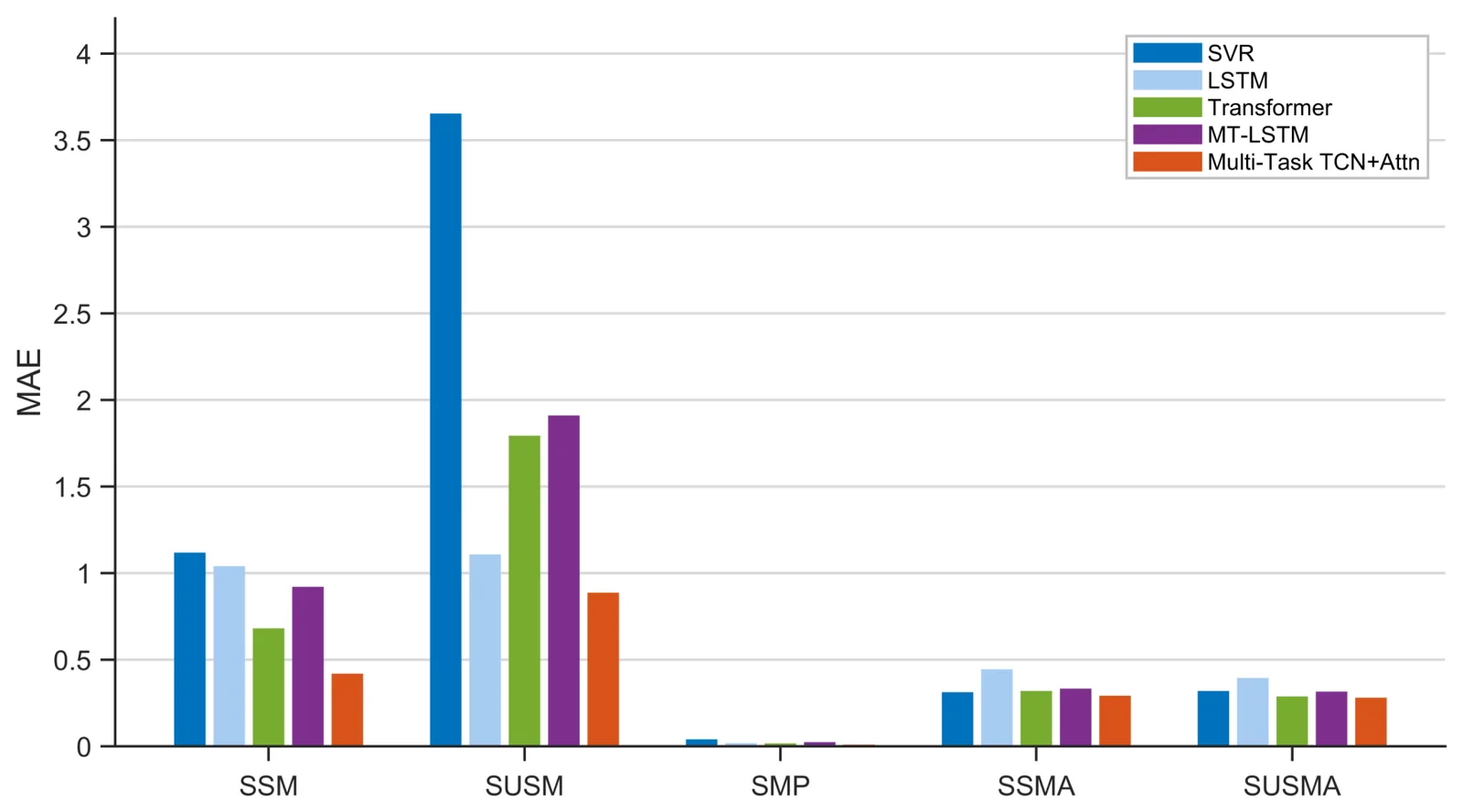
MAE comparison of MTC-TCN-Attn with representative benchmark models across five prediction targets.

Figure 9, Figure 10 and Figure 11 visually summarize the benchmark results and show that the proposed model maintains a consistent advantage across the different model families and prediction targets.

### 4.3. Temporal Robustness Under Different Chronological Splits

The temporal robustness experiment shows that model performance changes with the selected test period, but the proposed model retains strong predictive capability for the principal soil-moisture targets under alternative chronological partitions. Therefore, the performance observed in the main experiment is not restricted to a single train–test split, although the degree of improvement varies across years and targets.

In addition to the original chronological 80/20 split, two rolling chronological evaluations were conducted. Fold 1 used data from 2016–2019 for training and 2020 for testing, whereas Fold 2 used data from 2017–2020 for training and 2021 for testing. Only the training and testing periods were changed. The model architectures, input variables, 30-day historical window, one-step prediction horizon, optimization settings, and evaluation metrics were kept unchanged. For each fold, normalization statistics were recalculated exclusively from the corresponding training period. The evaluation settings for the original split and the two additional chronological splits are summarized in Table 9.

**Figure 12:**
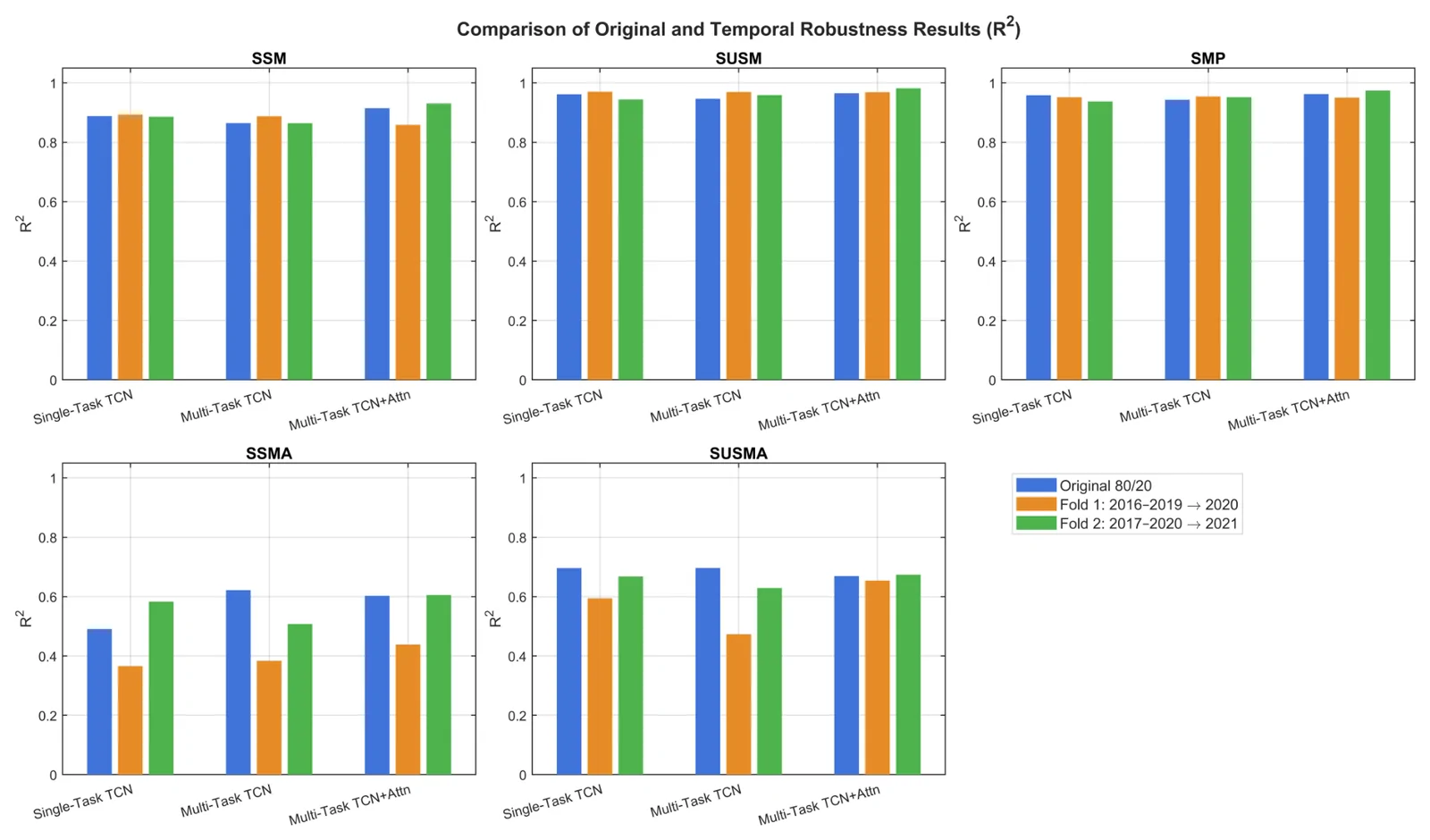
Comparison of $R^{2}$ under the original chronological 80/20 split and the two temporal robustness folds for the three TCN-based models across five prediction targets.

The results in Figure 12 show that the three principal targets retain relatively high predictive accuracy across the different temporal settings. For MTC-TCN-Attn, the $R^{2}$ values of SSM are 0.9147, 0.8586, and 0.9307 under the original split, Fold 1, and Fold 2, respectively. The corresponding values for SUSM are 0.9651, 0.9689, and 0.9817, while those for SMP are 0.9622, 0.9505, and 0.9745. In particular, SUSM and SMP maintain $R^{2}$ values above 0.95 in all three settings, demonstrating relatively stable predictive performance across different test periods.

**Figure 13:**
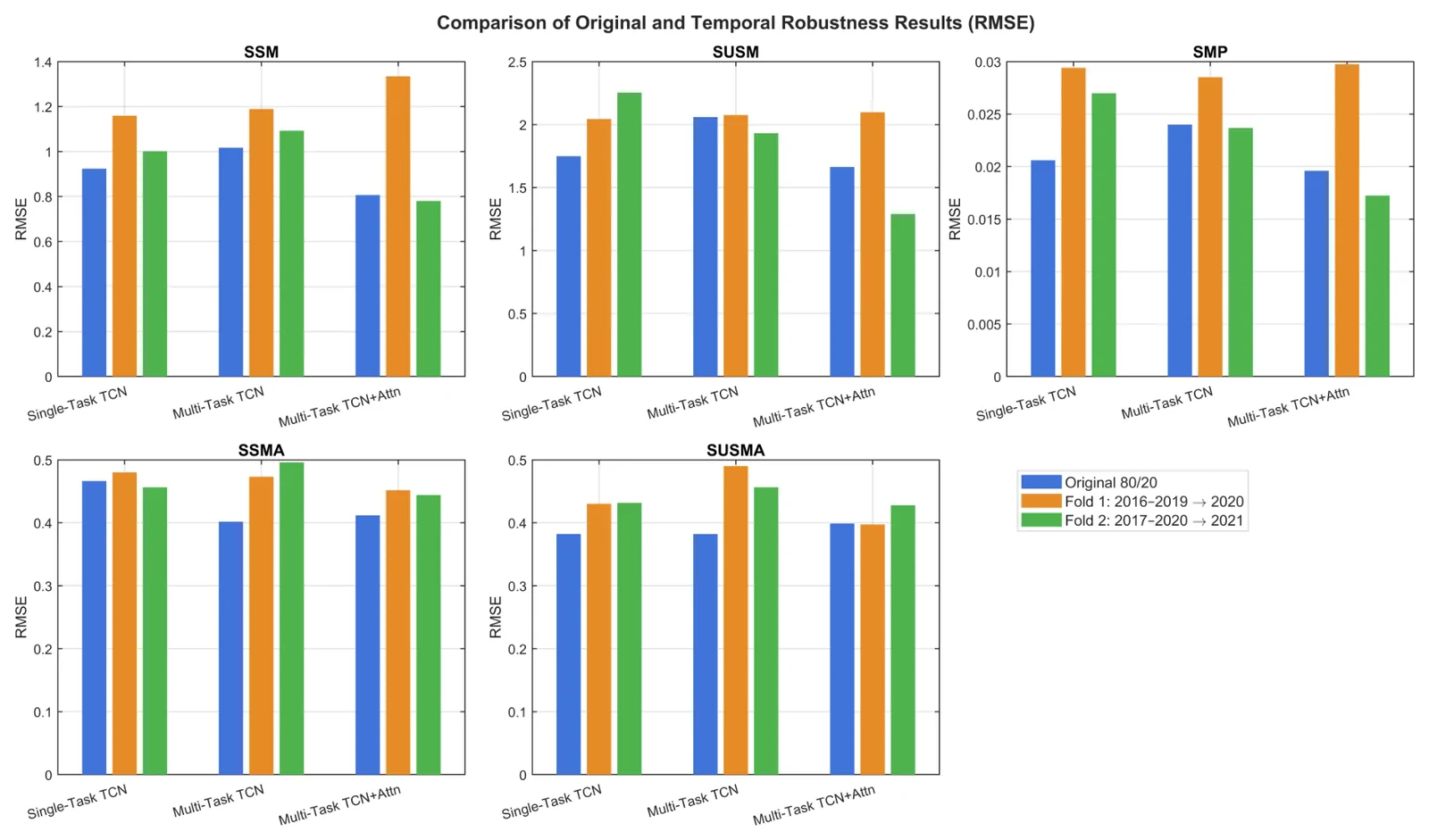
Comparison of RMSE under the original chronological 80/20 split and the two temporal robustness folds for the three TCN-based models across five prediction targets.

The RMSE results in Figure 13 show a similar pattern. Although Fold 1 produces larger errors for several targets, MTC-TCN-Attn achieves substantially lower RMSE values in Fold 2, reaching 0.7803, 1.2904, and 0.0172 for SSM, SUSM, and SMP, respectively. These values are lower than those obtained under the original split for the same three targets.

**Figure 14:**
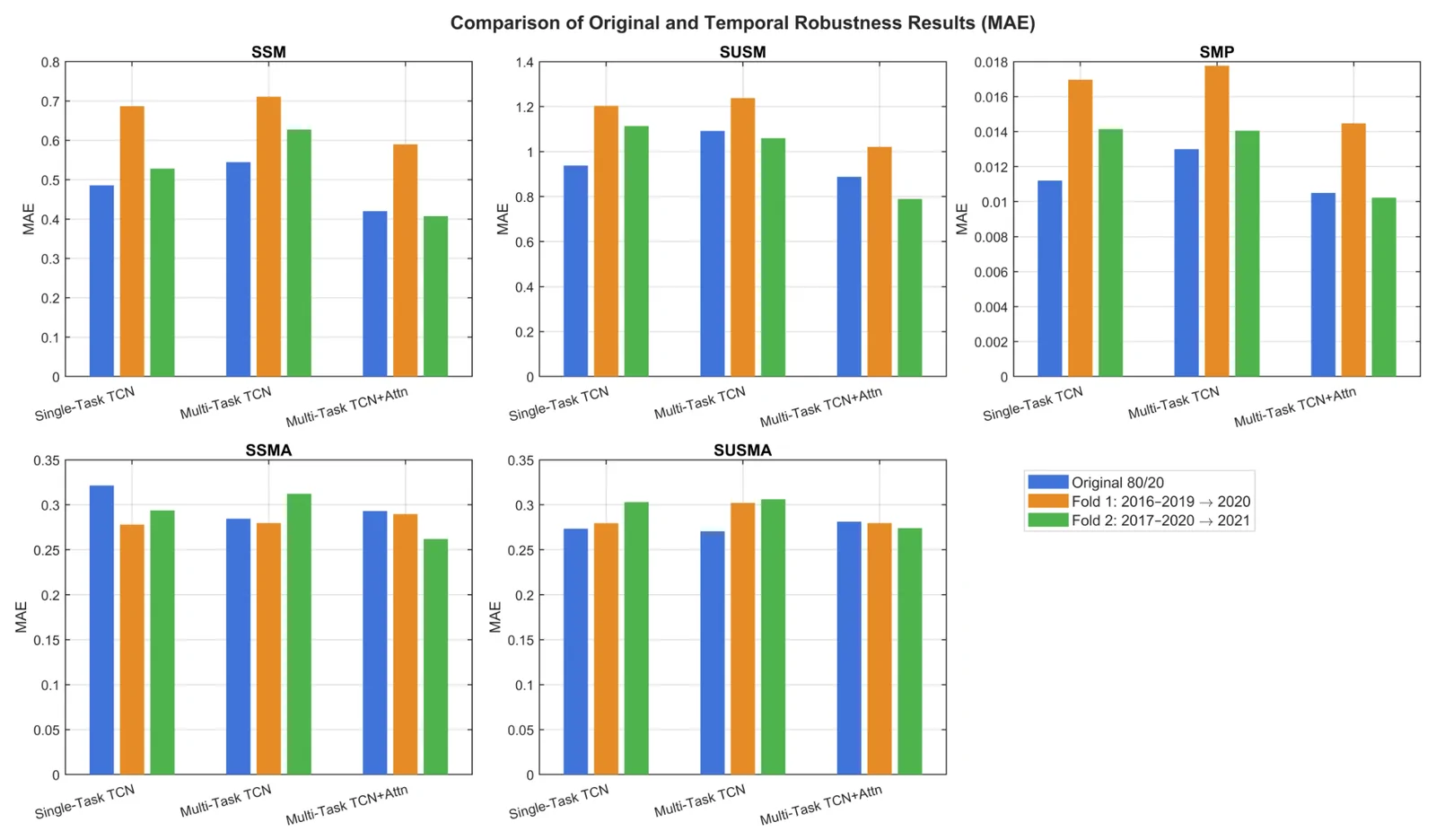
Comparison of MAE under the original chronological 80/20 split and the two temporal robustness folds for the three TCN-based models across five prediction targets.

The MAE comparison in Figure 14 further confirms this pattern. In Fold 2, MTC-TCN-Attn obtains the lowest MAE values among the three TCN-based models for all five prediction targets. The results of Fold 1, however, also show that the relative model ranking is not completely invariant to the test period, particularly for SSM and the anomaly-based targets.

Overall, the temporal experiments provide two complementary findings. First, the absolute evaluation metrics vary across different test years, indicating sensitivity to temporal distribution changes. Second, the proposed model continues to maintain strong predictive performance for the principal targets, particularly SUSM and SMP, and achieves the strongest overall results in Fold 2. Thus, the main performance advantage of MTC-TCN-Attn is not confined to the original chronological split, although its magnitude depends on the temporal evaluation period.

### 4.4. Gradient Conflict Analysis

The nonuniform performance gains observed in the previous experiments suggest that the five prediction tasks may not be equally compatible during joint optimization. To directly examine this issue, gradient conflict analysis was conducted for the Multi-Task TCN and MTC-TCN-Attn models. The results reveal clear optimization heterogeneity among the five tasks and provide direct evidence that positive and negative transfer can coexist within the shared multi-task framework.

For each mini-batch, the gradients generated by individual task losses with respect to the shared model parameters were calculated separately. The pairwise gradient cosine similarity between tasks *i* and *j* is defined as

(27) $$C_{ij}=\frac{g_{i}^{T}g_{j}}{∥g_{i}{∥}_{2}{∥g_{j}∥}_{2}},$$

where $g_{i}$ and $g_{j}$ denote the gradients of tasks *i* and *j* with respect to the shared parameters. A positive value indicates locally aligned optimization directions, whereas a negative value indicates gradient conflict. The corresponding pairwise conflict rate is defined as

(28) $$R_{ij}=\frac{N(C_{ij}<0)}{N_{\mathrm{batch}}}\times 100\%,$$

where $N(C_{ij}<0)$ denotes the number of analyzed mini-batches in which the corresponding task pair exhibits negative cosine similarity.

Table 10 summarizes the overall and task-level gradient statistics.

**Figure 15:**
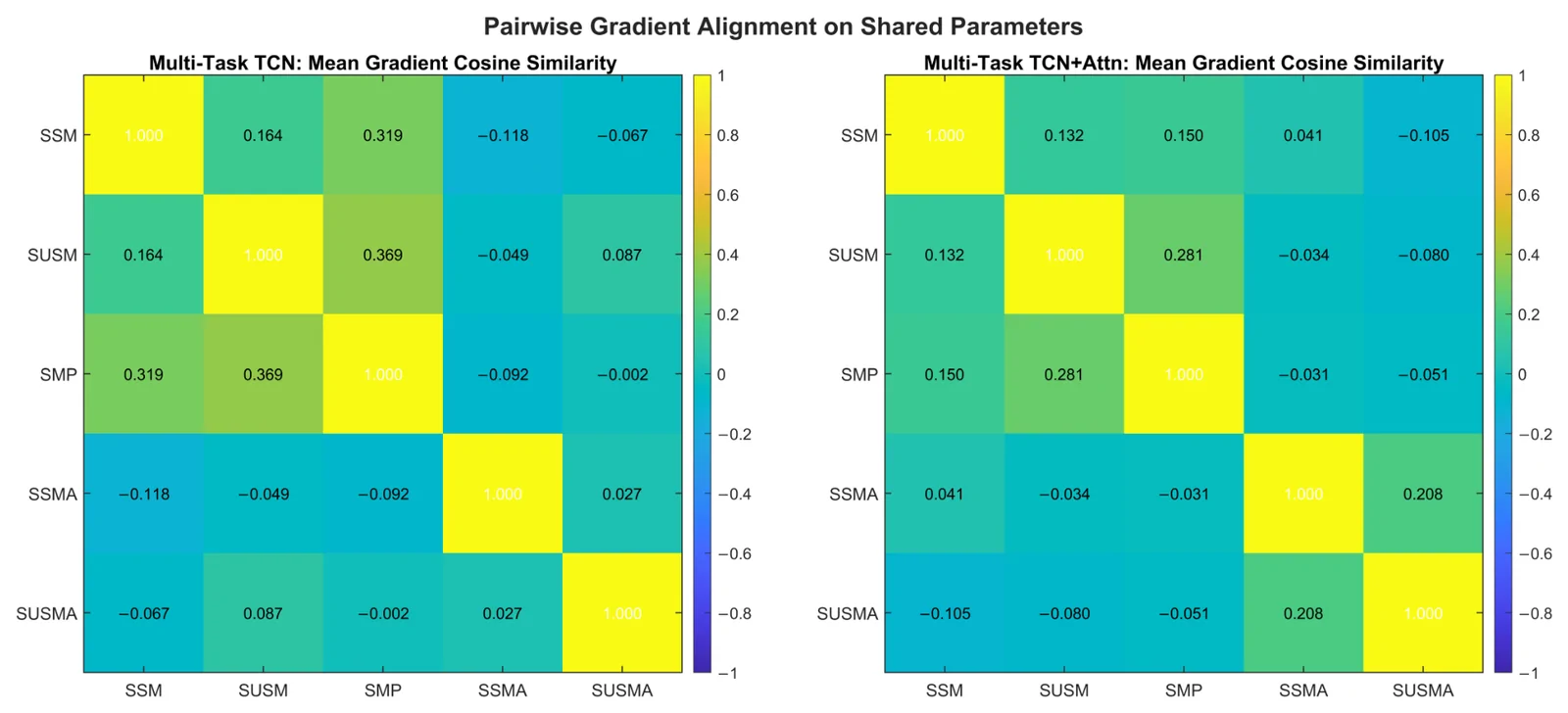
Pairwise mean gradient cosine similarity on shared parameters for the Multi-Task TCN and MTC-TCN-Attn models. Positive values indicate aligned gradient directions, whereas negative values indicate gradient conflict.

**Figure 16:**
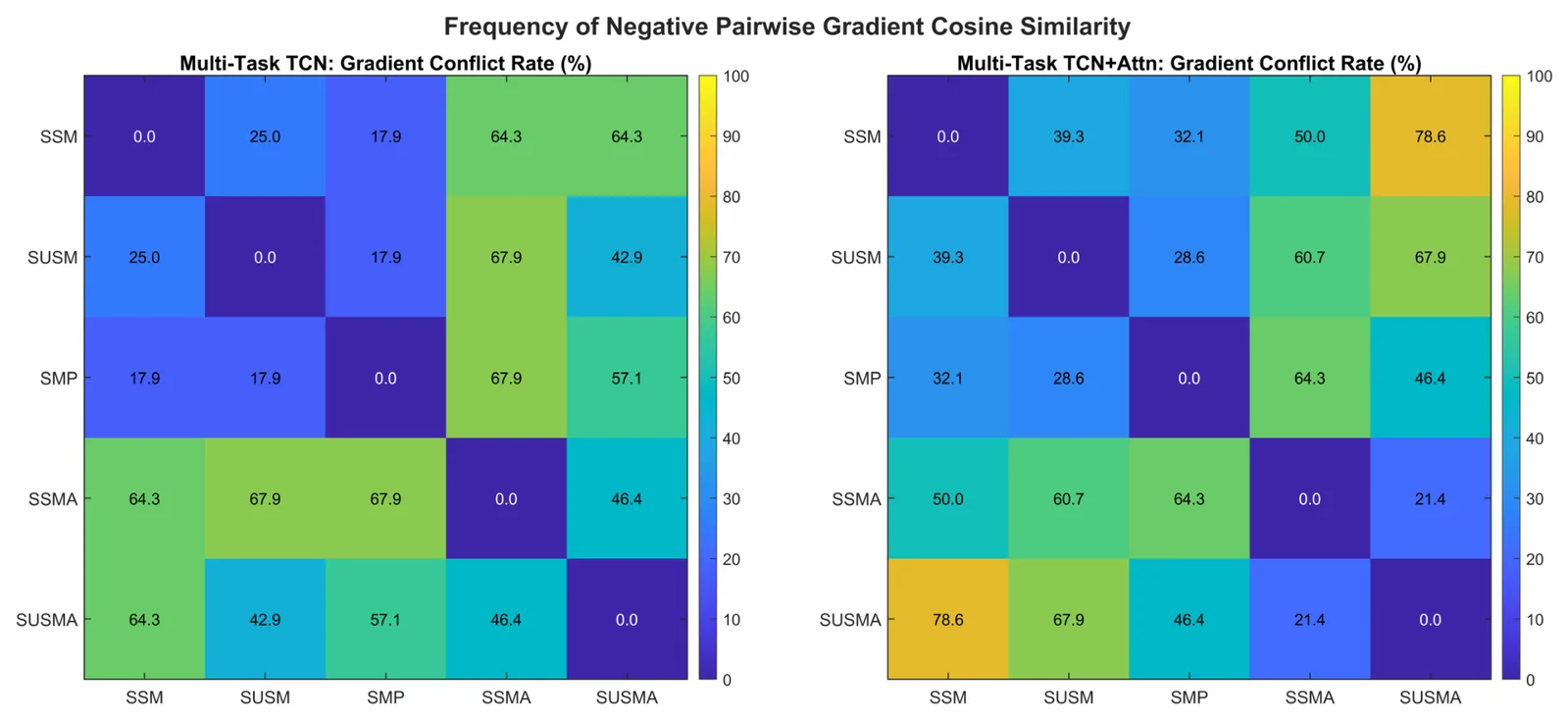
Pairwise gradient conflict rates for the Multi-Task TCN and MTC-TCN-Attn models. The conflict rate represents the percentage of analyzed mini-batches with negative pairwise gradient cosine similarity.

The pairwise mean gradient cosine similarities are illustrated in Figure 15, while the corresponding gradient conflict rates are presented in Figure 16.

The pairwise results reveal a particularly clear distinction between the principal soil-moisture targets and the anomaly-based tasks. In the Multi-Task TCN, SSM, SUSM, and SMP show positive pairwise mean cosine similarities of 0.164, 0.319, and 0.369, with relatively low conflict rates ranging from 17.9% to 25.0%. This indicates that these three tasks frequently generate mutually compatible optimization directions on the shared parameters.

In contrast, SSMA exhibits negative mean gradient cosine similarities with SSM, SUSM, and SMP, with values of −0.118, −0.049, and −0.092, respectively. Their corresponding conflict rates exceed 64%. The optimization behavior of the anomaly-based targets is therefore substantially less consistent with that of the principal soil-moisture targets. This gradient-level evidence is consistent with the task-dependent performance changes observed in the internal multi-task comparison.

The introduction of self-attention changes the distribution of task interactions rather than uniformly reducing gradient conflict. For example, the mean cosine similarity of SSM–SSMA improves from -0.118 to 0.041, while that of SSMA–SUSMA increases from 0.027 to 0.208. Correspondingly, the conflict rates of these two pairs decrease from 64.3% to 50.0% and from 46.4% to 21.4%, respectively.

However, several other task pairs show increased conflict. The conflict rate of SSM–SUSMA increases from 64.3% to 78.6%, SUSM–SUSMA increases from 42.9% to 67.9%, and SSM–SUSM increases from 25.0% to 39.3%. At the overall level, the mean gradient cosine similarity changes from 0.0638 to 0.0514, while the conflict rate changes from 47.14% to 48.93%.

These results demonstrate that the five prediction tasks are not uniformly cooperative during shared optimization. Instead, distinct task pairs exhibit substantially different degrees of gradient alignment and conflict. Self-attention modifies these interactions but does not eliminate conflict globally. The gradient experiment therefore provides direct optimization-level evidence for the heterogeneous performance gains observed in the multi-task prediction results.

## 5. Discussion

### 5.1. Overall Predictive Performance and Model Comparison

Soil moisture dynamics are jointly affected by precipitation, evapotranspiration, temperature, interlayer water transfer, and antecedent moisture conditions, resulting in pronounced nonlinearity, lagged responses, and interannual nonstationarity. Although different soil moisture indicators share some physical drivers, they have distinct statistical meanings and temporal dynamics. Conventional single-task models optimize each target independently but cannot exploit complementary information across variables. In contrast, multi-task learning facilitates cross-task knowledge transfer through shared representations, thereby reducing redundant modeling and potentially improving generalization when training data are limited. Previous studies have shown that jointly forecasting related soil moisture targets or multiple forecast horizons can outperform independent modeling [42], while attention mechanisms can help identify the predictors and historical time steps that are most informative for soil moisture prediction [27]. However, the benefits of multi-task learning depend on task relatedness, and excessive sharing may cause tasks to interfere with one another in the shared parameter space, leading to negative transfer [23,24]. Accordingly, this study compared single-task and multi-task prediction performance and further examined the optimization relationships among the targets through a gradient-conflict experiment to explain both the performance gains and the task-dependent behavior of MTC-TCN-Attn.

The external baseline experiments first confirmed the overall effectiveness of the proposed method. Compared with SVR, LSTM, Transformer, and Multi-Task Long Short-Term Memory (MT-LSTM), MTC-TCN-Attn achieved the highest $R^{2}$ and the lowest RMSE and MAE for all five targets. Relative to the strongest external baseline for each target and metric, MTC-TCN-Attn produced absolute $R^{2}$ gains of 0.020–0.221, RMSE reductions of 12.6–47.3%, and MAE reductions of 2.6–40.8%. Although MT-LSTM also performs joint prediction, it was consistently outperformed by MTC-TCN-Attn, indicating that the observed gains cannot be attributed solely to the use of multi-task learning. Rather, they arise from the combination of the sharing strategy and the temporal feature-extraction architecture. Previous multi-task soil moisture research has mainly treated different forecast horizons as related tasks and demonstrated the benefit of joint learning for 24–48 h forecasts [42]. The present study extends this setting to five heterogeneous soil moisture indicators with different physical meanings and statistical properties. It combines multi-scale causal convolution and self-attention within a shared TCN encoder and explicitly analyzes cross-task cooperation and competition through task-gradient statistics. Compared with existing multi-task studies, the contribution of the proposed method therefore lies not only in extending homogeneous multi-horizon forecasting to heterogeneous multi-variable prediction, but also in explicitly evaluating negative transfer and generalization across temporal splits.

### 5.2. Multi-Task Learning Effects and Anomaly Variable Performance

The ablation results further demonstrate that the effect of multi-task sharing is strongly task dependent. When Single-Task TCN was replaced by Multi-Task TCN, the $R^{2}$ values for SSM, SUSM, and SMP decreased from 0.8882, 0.9614, and 0.9583 to 0.8645, 0.9465, and 0.9431, respectively. In contrast, the $R^{2}$ for SSMA increased from 0.4905 to 0.6216, whereas that for SUSMA changed only marginally from 0.6961 to 0.6963. SSM, SUSM, and SMP directly characterize soil moisture states and exhibit relatively strong temporal continuity, but variables representing different depths or different expressions of moisture conditions are not equally sensitive to local fluctuations, lagged responses, and long-term trends. SSMA and SUSMA, by comparison, describe anomalous departures from reference conditions and therefore have variability and statistical distributions that differ from those of the original moisture-state variables. Under hard parameter sharing, all five tasks update the same feature-extraction parameters. Related tasks can exchange useful information, but tasks with different feature requirements must also compete for the limited capacity of the shared representation. Consequently, Multi-Task TCN produced both positive and negative transfer rather than uniformly improving every target.

The gradient-conflict experiment provides direct evidence for this negative-transfer mechanism. In Multi-Task TCN, the mean gradient cosine similarities for SSM–SUSM, SSM–SMP, and SUSM–SMP were 0.164, 0.319, and 0.369, respectively, with corresponding conflict rates of 25.0%, 17.9%, and 17.9%. These results indicate that the optimization directions of the three primary moisture-state tasks were generally aligned. By contrast, the mean gradient cosine similarities between SSMA and SSM, SUSM, and SMP were −0.118, −0.049, and −0.092, respectively, and the corresponding conflict rates reached 64.3%, 67.9%, and 67.9%. The conflict rate between SSM and SUSMA was also 64.3%. When two task gradients point in opposing directions, an update that reduces the loss of one task may increase the loss of the other, which is a typical form of gradient interference in multi-task optimization. Yu et al. [25] similarly showed that conflicting gradients can prevent a multi-task model from simultaneously following descent directions favorable to all tasks, while Lee et al. [24] demonstrated that symmetric sharing among tasks with different levels of relatedness can induce negative transfer. Consistent with these findings, the strong gradient competition between the anomaly tasks and the primary moisture-state tasks provides an optimization-based explanation for why Multi-Task TCN underperformed Single-Task TCN on SSM, SUSM, and SMP.

The improvement or stability of SSMA and SUSMA under Multi-Task TCN does not contradict the gradient-conflict results. Gradient conflict characterizes the local alignment of task-specific optimization directions in the shared parameter space, whereas predictive performance reflects the combined effects of beneficial knowledge transfer and optimization interference. SSMA was relatively difficult to predict in the single-task setting, with an $R^{2}$ of only 0.4905. During joint training, it could exploit the basic moisture-state and temporal-change information learned from SSM, SUSM, and SMP. The resulting positive transfer outweighed the detrimental effect of gradient competition, increasing the $R^{2}$ for SSMA to 0.6216. The much smaller change observed for SUSMA indicates that the two anomaly variables do not have identical relationships with the primary tasks. Thus, the main advantage of multi-task modeling over single-task modeling is its ability to exploit complementary information across targets; however, this advantage is conditional, and strong task heterogeneity may cause parameter sharing to sacrifice the task-specific optimum for some outputs.

After self-attention was added to Multi-Task TCN, the $R^{2}$ values for SSM, SUSM, and SMP increased to 0.9147, 0.9651, and 0.9622, respectively. These improvements not only recovered the losses caused by simple multi-task sharing but also surpassed the corresponding Single-Task TCN results. In contrast, the $R^{2}$ values for SSMA and SUSMA decreased from 0.6216 and 0.6963 to 0.6026 and 0.6692, respectively. Unlike the three moisture-state variables, SSMA and SUSMA represent departures from a calendar-period climatology rather than moisture states themselves. Because this climatological reference is not explicitly included among the model inputs, their variations may be harder to infer from the same 30-day history. Moreover, attention operates on temporal features shared by all five tasks; time steps that improve predictions of SSM, SUSM, and SMP may not be equally informative for the two anomaly targets. These factors may partly explain the modest decreases in their $R^{2}$ values. This pattern indicates that the primary role of self-attention is not to provide an equal gain for all tasks, but to improve the suitability of the shared temporal representation for particular targets. TCN extracts multi-scale temporal features through dilated causal convolutions, efficiently enlarging its receptive field while preserving temporal causality [26]. Nevertheless, the importance of individual historical time steps is still encoded implicitly by convolutional parameters. Self-attention establishes global relationships across the input window and dynamically reallocates weights among time steps according to the current sequence [28]. For SSM, SUSM, and SMP, which exhibit relatively strong continuity and temporal dependence, this mechanism can emphasize informative antecedent states and lagged moisture responses, thereby improving prediction accuracy. Li et al. [27] likewise reported that temporal and predictor attention improved soil moisture prediction and model interpretability. Wang et al. [17] further showed that the effectiveness of attention depends on the prediction task and model structure: feature attention produced relatively stable gains, whereas temporal attention offered limited benefits or even degraded performance in some settings. These findings are consistent with the present observation that the primary moisture variables benefited from self-attention while the anomaly variables did not improve synchronously.

The gradient results further indicate that the improvement achieved by MTC-TCN-Attn primarily arose from enhanced temporal representation rather than from the elimination of gradient conflicts. After self-attention was introduced, the overall mean gradient cosine similarity decreased from 0.0638 to 0.0514, while the overall conflict rate increased slightly from 47.14% to 48.93%. The mean cosine similarities for SSM–SUSM, SSM–SMP, and SUSM–SMP also did not increase. Nevertheless, the predictive performance of all three primary moisture-state tasks improved, demonstrating that higher-quality temporal features can enhance prediction even when cross-task gradient alignment does not improve. For SUSMA, the conflict rates for SSM–SUSMA and SUSM–SUSMA increased from 64.3% and 42.9% to 78.6% and 67.9%, respectively, and this stronger optimization competition was consistent with the observed performance degradation. For SSMA, the task-level mean cosine similarity increased from −0.058 to 0.046 and the mean conflict rate decreased from 61.61% to 49.11%, yet its $R^{2}$ still declined slightly. This result confirms that gradient conflict is not the sole determinant of predictive performance; the intrinsic temporal structure, noise level, and compatibility of each target with the shared representation are also important. A more accurate mechanistic interpretation is, therefore, that self-attention enhances global temporal-dependency modeling while reorganizing interactions among tasks in the shared space, rather than uniformly alleviating negative transfer across all task pairs.

### 5.3. Model Robustness Under Different Temporal Splits

The temporal robustness experiments also show why differences in absolute performance across temporal splits are reasonable. The original experiment used a chronological 80%/20% split of the consecutive samples, whereas Fold 1 was trained on data from 2016–2019 and tested on the complete year 2020, and Fold 2 was trained on data from 2017–2020 and tested on the complete year 2021. These three settings differ in their training periods, test periods, and target distributions. Moreover, soil moisture is affected by interannual meteorological variability, seasonal processes, and extreme wetting or drying events. Therefore, identical numerical results should not be expected across the three splits even when the model architecture and training procedure remain unchanged. The larger difference between Fold 1 and the original experiment may mainly reflect the replacement of the original consecutive holdout segment with an independent full-year test set for 2020, which can produce a more pronounced distribution shift between training and testing. Fold 2 incorporated data through 2020 during training and, consequently, learned a different and more recent set of moisture dynamics, so its relative performance in 2021 was also expected to differ from that of Fold 1. This explanation attributes the differences to interannual distribution shifts in the time series rather than assigning them to any single meteorological factor without direct evidence.

The evaluation metrics also respond differently to such distribution shifts. In Fold 1, MTC-TCN-Attn achieved the lowest MAE for SSM, SUSM, and SMP, although it did not obtain the best RMSE and $R^{2}$ for every one of these targets. This pattern suggests that the model maintained relatively small absolute errors for most samples, while a limited number of larger residuals or changes in the variance of the test targets may have had a stronger effect on RMSE and $R^{2}$. In Fold 2, MTC-TCN-Attn achieved the best $R^{2}$, RMSE, and MAE for all five tasks. More importantly, when the two year-based holdout folds were averaged, MTC-TCN-Attn obtained higher mean $R^{2}$ values and lower mean RMSE and MAE values than both Single-Task TCN and Multi-Task TCN for every target. Thus, although different years caused local variations in absolute performance and model ranking, the main conclusion that the proposed model performs better overall than the two TCN baselines remained valid in the aggregated results.

Overall, the experiments under different temporal partitions show that the absolute predictive performance of the models varies to some extent with the selected training and testing periods, reflecting distributional differences in soil moisture time series across years. Nevertheless, MTC-TCN-Attn maintained relatively high predictive accuracy for the principal soil moisture variables in both year-based holdout evaluations, and its mean performance across the two year-based folds was generally better than that of the Single-Task TCN and Multi-Task TCN. These results indicate that the performance observed under the original 80%/20% split is not determined solely by a single temporal partition, and that the proposed model exhibits good temporal robustness across different evaluation periods at the investigated location. At the same time, the observed performance variations across years also highlight the importance of considering temporal distribution changes when evaluating soil moisture time-series prediction models.

### 5.4. Model Applicability and Study Limitations

From the perspective of model architecture and input formulation, MTC-TCN-Attn is mainly designed for joint prediction of multi-variate time series with continuous historical observations of meteorological variables and soil moisture. The framework employs a TCN to extract historical features at different temporal scales, uses self-attention to further model global dependencies among key time steps, and adopts multi-task learning to simultaneously predict multiple interrelated soil-moisture-related variables. Therefore, when other study regions provide meteorological and soil moisture time series with the same or similar variable definitions, the same modeling framework can be retrained and evaluated for those regions.

However, the dataset used in the present study was obtained from a single spatial grid located in the Jodhpur region of Rajasthan, India, at 26.3° N, 72.5° E, covering the period from 2016 to 2021. Therefore, the current experiments mainly evaluate the predictive performance of the model at this study location and its stability across different temporal periods. The original chronological 80%/20% split together with the two full-year holdout experiments provides further evaluation of model performance from the temporal perspective, whereas the present data conditions are still insufficient for a comprehensive assessment of model transferability across different geographical regions.

Different regions may differ substantially in precipitation and temperature conditions, soil properties, cropping systems, and agricultural water-management practices. These differences may alter the statistical relationships and temporal response patterns between meteorological variables and soil moisture. Therefore, the results obtained in the Jodhpur region should not be directly interpreted as evidence that the model will achieve the same predictive performance across all climatic regions, soil types, and agricultural growing conditions. The current results mainly demonstrate that MTC-TCN-Attn can effectively exploit information among multiple variables and historical temporal dependencies under the investigated data conditions, while its cross-regional applicability still requires further validation using data from additional independent regions.

Future work will further improve the present study in two main directions. First, from the perspective of multi-task learning, dynamic task weighting, task-specific branches, or gradient-correction strategies can be introduced to preserve the benefits of cross-task knowledge sharing while further reducing negative transfer between the anomaly variables and the principal soil-moisture-state variables. Second, for model applicability assessment, future studies will incorporate multiple independent spatial grids or study regions covering different climatic conditions, soil types, and agricultural growing environments. Spatial block validation, leave-one-location-out validation, and independent cross-regional testing will also be considered to further evaluate the spatial transferability and cross-regional applicability of the proposed model.

Overall, the experimental results show that multi-task learning can exploit complementary information among multiple soil moisture prediction targets through shared representations, while differences in task relatedness and optimization directions also lead to clear task-dependent effects. Self-attention improves the temporal feature representation of SSM, SUSM, and SMP, but does not produce simultaneous improvements for SSMA and SUSMA, nor does it uniformly eliminate gradient conflicts among different tasks. The experiments under different temporal partitions further demonstrate that the model maintains good temporal predictive stability at the investigated study location. In future work, more flexible multi-task optimization strategies together with independent multi-region validation can further improve the applicability of the proposed method under complex agricultural conditions.

## 6. Conclusions

This paper investigates the multi-target collaborative prediction of wheat soil moisture and proposes a multi-task learning framework, termed MTC-TCN-Attn, which integrates a Temporal Convolutional Network (TCN) with a multi-head self-attention mechanism. By combining temporal feature extraction and adaptive temporal dependency modeling, the proposed framework aims to address the limitations of conventional soil moisture prediction approaches in capturing long-term dependencies and exploiting correlations among multiple soil-moisture-related variables.

A multi-task prediction architecture was established based on a shared TCN feature extraction backbone, where causal convolution, dilated convolution, and residual connections were employed to extract multi-scale temporal representations. Furthermore, a multi-head self-attention module was introduced after the TCN encoder to dynamically assign weights to informative time steps and enhance the representation of long-range temporal dependencies. To comprehensively evaluate the effectiveness of the proposed framework, we first established three progressively developed model variants, namely, Single-Task TCN, Multi-Task TCN, and MTC-TCN-Attn, to separately examine the effects of multi-task learning and self-attention. We further conducted external benchmark comparisons with SVR, LSTM, Transformer, and Multi-Task Long Short-Term Memory (MT-LSTM), temporal robustness experiments under different chronological data partitions, and a task-gradient conflict analysis.

The experimental results demonstrate that the proposed MTC-TCN-Attn model achieves superior prediction performance for the primary physical soil moisture variables, including SSM, SUSM, and SMP, obtaining $R^{2}$ values of 0.9147, 0.9651, and 0.9622, respectively. Compared with both the single-task TCN and multi-task TCN models, the proposed model achieves lower RMSE and MAE values, indicating improved prediction accuracy and robustness. Further analysis reveals that the multi-task learning strategy effectively exploits the inherent correlations among different soil-moisture-related variables through shared feature representations, thereby improving the generalization capability of the model. Meanwhile, the integration of the self-attention mechanism further enhances prediction performance by adaptively identifying critical temporal information and compensating for the limitation of TCN in dynamically weighting different time steps. Beyond the ablation comparisons with Single-Task TCN and Multi-Task TCN, the proposed model was further evaluated against representative machine learning and deep learning baselines, including SVR, LSTM, Transformer, and Multi-Task Long Short-Term Memory (MT-LSTM). MTC-TCN-Attn achieved the highest $R^{2}$ and the lowest RMSE and MAE for all five prediction targets; relative to the strongest external baseline for each target and metric, it produced absolute $R^{2}$ gains of 0.020–0.221, RMSE reductions of 12.6–47.3%, and MAE reductions of 2.6–40.8%. Compared with existing multi-task soil moisture studies that mainly formulate different forecast horizons of the same variable as related tasks, the proposed framework jointly models heterogeneous moisture-state and anomaly variables by integrating multi-scale TCN feature extraction with self-attention, and its overall advantage was further supported by the chronological holdout experiments.

Overall, the proposed MTC-TCN-Attn framework provides an effective solution for joint prediction of multiple soil moisture variables by jointly modeling temporal dependencies and cross-task correlations. The proposed method improves the representation capability of complex soil moisture dynamics and provides a promising modeling approach for intelligent irrigation management, precision agriculture, and soil water resource optimization. Future studies will focus on incorporating additional environmental variables and exploring more adaptive multi-task optimization strategies to further improve model transferability and applicability under diverse agricultural conditions.

## Figures and Tables

**Table 1:** Data sources, roles, units, and definitions of the variables used in this study.

Variable	Full Name	Source	Role	Unit	Description
T2MDEW	2 m Dew Point Temperature	NASA POWER	Input	°C	Dew point temperature at 2 m above the surface.
PRECTOTCORR	Corrected Precipitation	NASA POWER	Input	mm/day	Daily corrected precipitation amount.
WD10M	10 m Wind Direction	NASA POWER	Input	degree	Wind direction at 10 m above the surface.
TS	Earth Skin Temperature	NASA POWER	Input	°C	Temperature of the land surface skin.
T2M	2 m Air Temperature	NASA POWER	Input	°C	Air temperature at 2 m above the surface.
SSM	Surface Soil Moisture	NASA–USDA SMAP	Input/ Output	mm	Modeled water storage in the surface layer of the modified two-layer Palmer model.
SUSM	Subsurface Soil Moisture	NASA–USDA SMAP	Input/Output	mm	Modeled water storage in the subsurface/ root-zone layer of the modified two-layer Palmer model.
SMP	Soil Moisture Profile	NASA–USDA SMAP	Output	fraction	Dimensionless soil-moisture-profile fraction ranging approximately from 0 to 1, representing the relative moisture status of the modeled soil profile.
SSMA	Surface Soil Moisture Anomaly	NASA–USDA SMAP	Output	dimensionless	Standardized anomaly of surface soil moisture relative to its climatological condition.
SUSMA	Subsurface Soil Moisture Anomaly	NASA–USDA SMAP	Output	dimensionless	Standardized anomaly of subsurface soil moisture relative to its climatological condition.

**Table 2:** Detailed comparison of the three comparative models.

Comparative Dimension	M1 Single-Task TCN	M2 Multi-Task TCN	M3 MTC-TCN-Attn
Task setting	Single-task independent training	Joint multi-task training	Joint multi-task training
Backbone	TCN	TCN	TCN
Attention	None	None	4-head self-attention
Parameter sharing	No	Hard parameter sharing	Hard parameter sharing
Output	1 target per model	5 targets	5 targets
Loss	Single-task MSE	Equal-weight multi-task MSE	Equal-weight multi-task MSE
Trainable parameters	105,089	105,349	109,685
Number of models	5	1	1

**Table 3:** $R^{2}$ values of the three TCN-based models across five prediction targets.

Model	SSM	SUSM	SMP	SSMA	SUSMA
Single-Task TCN	0.8882	0.9614	0.9583	0.4905	0.6961
Multi-Task TCN	0.8645	0.9465	0.9431	**0.6216**	**0.6963**
MTC-TCN-Attn	**0.9147**	**0.9651**	**0.9622**	0.6026	0.6692

*Note:* Bold values indicate the highest $R^{2}$ for each prediction target.

**Table 4:** RMSE values of the three TCN-based models across five prediction targets.

Model	SSM	SUSM	SMP	SSMA	SUSMA
	(mm)	(mm)	(-)	(-)	(-)
Single-Task TCN	0.9240	1.7489	0.0206	0.4664	0.3822
Multi-Task TCN	1.0172	2.0595	0.0240	**0.4019**	**0.3821**
MTC-TCN-Attn	**0.8069**	**1.6631**	**0.0196**	0.4119	0.3988

*Note:* Bold values indicate the lowest RMSE for each prediction target.

**Table 5:** MAE values of the three TCN-based models across five prediction targets.

Model	SSM	SUSM	SMP	SSMA	SUSMA
	(mm)	(mm)	(-)	(-)	(-)
Single-Task TCN	0.4856	0.9381	0.0112	0.3214	0.2734
Multi-Task TCN	0.5447	1.0920	0.0130	**0.2845**	**0.2706**
MTC-TCN-Attn	**0.4201**	**0.8879**	**0.0105**	0.2931	0.2813

*Note:* Bold values indicate the lowest MAE for each prediction target.

**Table 6:** $R^{2}$ Comparison with representative benchmark models.

Model	SSM	SUSM	SMP	SSMA	SUSMA
SVR	0.6853	0.7047	0.7008	0.4081	0.4728
LSTM	0.5111	0.9448	0.8858	0.0328	0.3257
Transformer (NoPE)	0.6934	0.7703	0.8968	0.3976	0.5671
MT-LSTM	0.6454	0.8508	0.8173	0.4125	0.5495
MTC-TCN-Attn	**0.9147**	**0.9651**	**0.9622**	**0.6026**	**0.6692**

*Note:* Bold values indicate the highest $R^{2}$ for each prediction target among the compared models.

**Table 7:** RMSE comparison with representative benchmark models.

Model	SSM	SUSM	SMP	SSMA	SUSMA
	(mm)	(mm)	(-)	(-)	(-)
SVR	1.5499	4.8370	0.0551	0.5027	0.5034
LSTM	1.9320	2.0911	0.0340	0.6426	0.5693
Transformer (NoPE)	1.5299	4.2661	0.0324	0.5071	0.4562
MT-LSTM	1.6454	3.4381	0.0430	0.5008	0.4654
MTC-TCN-Attn	**0.8069**	**1.6631**	**0.0196**	**0.4119**	**0.3988**

*Note:* Bold values indicate the lowest RMSE for each prediction target among the compared models.

**Table 8:** MAE comparison with representative benchmark models.

Model	SSM	SUSM	SMP	SSMA	SUSMA
	(mm)	(mm)	(-)	(-)	(-)
SVR	1.1191	3.6543	0.0409	0.3132	0.3205
LSTM	1.0408	1.1088	0.0189	0.4457	0.3956
Transformer (NoPE)	0.6817	1.7943	0.0177	0.3204	0.2888
MT-LSTM	0.9216	1.9106	0.0248	0.3338	0.3167
MTC-TCN-Attn	**0.4201**	**0.8879**	**0.0105**	**0.2931**	**0.2813**

*Note:* Bold values indicate the lowest MAE for each prediction target among the compared models.

**Table 9:** Evaluation settings used in the temporal robustness experiment.

Setting	Training Period	Testing Period	Description
Original split	Chronological first 80%	Chronological last 20%	Main experiment setting
Fold 1	2016–2019	2020	4-year rolling split
Fold 2	2017–2020	2021	4-year rolling split

**Table 10:** Summary of gradient alignment and conflict statistics.

Task/Overall	Multi-Task TCN		MTC-TCN-Attn	
	Mean Cosine	Conflict Rate (%)	Mean Cosine	Conflict Rate (%)
Overall	0.0638	47.14	0.0514	48.93
SSM	0.0744	42.86	0.0547	50.00
SUSM	0.1428	38.39	0.0749	49.11
SMP	0.1484	40.18	0.0875	42.86
SSMA	−0.0580	61.61	0.0462	49.11
SUSMA	0.0115	52.68	−0.0067	53.57

## Data Availability

The data is available from the corresponding author upon reasonable request.
